# Identification of Genes and MicroRNAs Affecting Pre-harvest Sprouting in Rice (*Oryza sativa* L.) by Transcriptome and Small RNAome Analyses

**DOI:** 10.3389/fpls.2021.727302

**Published:** 2021-08-06

**Authors:** Minsu Park, Woochang Choi, Sang-Yoon Shin, Hongman Moon, Dowhan Lee, Yun-Shil Gho, Ki-Hong Jung, Jong-Seong Jeon, Chanseok Shin

**Affiliations:** ^1^Department of Agricultural Biotechnology, Seoul National University, Seoul, South Korea; ^2^Research Center for Plant Plasticity, Seoul National University, Seoul, South Korea; ^3^Graduate School of Biotechnology and Crop Biotech Institute, Kyung Hee University, Yongin, South Korea; ^4^Research Institute of Agriculture and Life Sciences, Seoul National University, Seoul, South Korea; ^5^Plant Genomics and Breeding Institute, Seoul National University, Seoul, South Korea

**Keywords:** rice (*Oryza sativa* L.), pre-harvest sprouting (PHS), dormancy, stress responses, transcriptome, small RNAome

## Abstract

Pre-harvest sprouting (PHS) is one of the primary problems associated with seed dormancy in rice (*Oryza sativa* L.). It causes yield loss and reduces grain quality under unpredictable humid conditions at the ripening stage, thus affecting the economic value of the rice crop. To resolve this issue, understanding the molecular mechanism underlying seed dormancy in rice is important. Recent studies have shown that seed dormancy is affected by a large number of genes associated with plant hormone regulation. However, understanding regarding the effect of heat stress on seed dormancy and plant hormones is limited. This study compared the transcriptome and small RNAome of the seed embryo and endosperm of two contrasting *japonica* rice accessions, PHS susceptible (with low seed dormancy) and PHS resistant (with high seed dormancy), at three different maturation stages. We found that 9,068 genes and 35 microRNAs (miRNAs) were differentially expressed in the embryo, whereas 360 genes were differentially expressed in the endosperm. Furthermore, we identified and verified the candidate genes associated with seed dormancy and heat stress-related responses in rice using quantitative real-time PCR. We newly discovered eight hormone-related genes, four heat shock protein-related genes, and two miRNAs potentially involved in PHS. These findings provide a strong foundation for understanding the dynamics of transcriptome and small RNAome of hormone- and heat stress-related genes, which affect PHS during seed maturation.

## Introduction

Rice (*Oryza sativa* L.) is one of the essential crops and the staple food for approximately 50% of the global population (Wei et al., 2013). In the rice field, pre-harvest sprouting (PHS) is crucial issue as it not only reduces the rice grain yield but also significantly affects the grain quality, resulting in a significant economic loss. Under normal conditions, rice seeds can be germinated by controlling their maturation. However, under excess moisture conditions during the period between seed maturation and harvest, the proper seed dormancy is broken, which causes PHS, an important issue that must be overcome to avoid the reduction in grain quality and yield (Gubler et al., 2005). Therefore, understanding the molecular basis of seed dormancy and maturation is crucial for controlling PHS.

Abscisic acid (ABA) plays an important role in the induction and maintenance of seed dormancy (Gubler et al., 2005; Finkelstein et al., 2008; Graeber et al., 2012; Shu et al., 2016). Abscisic aldehyde oxidase 3 (AAO3) catalyzes the final step of ABA biosynthesis. *Arabidopsis thaliana* mutants *aao3-2* and *aao3-3* and the rice mutant *osao3* exhibit lower seed dormancy and ABA levels than the corresponding wild type (González-Guzmán et al., 2004; Shi et al., 2021). In rice, the basic helix-loop-helix (bHLH) transcription factor gene *OsbHLH035* mediates seed germination. The *Osbhlh035* mutants showed delayed seed germination and up-regulated *OsAAO3* expression (Chen et al., 2018). ABA response and signaling are also involved in seed dormancy. GEM, a member of the GRAM (Glycosyltransferases, Rab-like GTPase Activators, Myotubularins) domain family proteins, is an ABA-responsive protein, and the *gem-1* mutant shows increased seed dormancy (Mauri et al., 2016). In rice, the overexpression of the F-box gene, *OsFbx352*, improves the germination rate of seeds when treated with ABA, and decreases the expression of ABA biosynthesis-related genes (*OsNced2* and *OsNced3*) (Frey et al., 2012; Song et al., 2012).

Ethylene, another plant hormone, promotes seed germination by counteracting ABA signaling (Arc et al., 2013; Corbineau et al., 2014). The metabolic precursor of ethylene, 1-aminocyclopropane-1-carboxylic acid (ACC), produced by ACC synthase (ACS), is oxidized by ACC oxidase (ACO) to form ethylene (Zarembinski and Theologis, 1994; Wang et al., 2002). Transgenic *Arabidopsis* lines overexpressing the bHLH transcription factor gene *PIF5* exhibit up-regulated *ACS4* expression and increased ethylene accumulation (Khanna et al., 2007). In addition, *aco2* mutants exhibit reduced seed germination via the ACC-mediated reversion of ABA (Linkies et al., 2009). In tomato (*Solanum lycopersicum*), overexpression of the *Ethylene Response Factor 2* (*SlERF2*) gene results in premature seed germination (Pirrello et al., 2006). On the other hand, the *Arabidopsis* mutant *aterf7* exhibits delayed seed germination and increased ABA sensitivity during seed germination (Song et al., 2005).

In addition to plant hormones, heat shock proteins (HSPs) affect seed dormancy and temperature. Plants synthesize HSPs under high temperature conditions (Kotak et al., 2007; Xue et al., 2014; Muthusamy et al., 2017). Moreover, plants overexpressing *HSPs* exhibit enhanced heat stress tolerance (Chauhan et al., 2012; Mu et al., 2013; Kaur et al., 2015). In *Arabidopsis* and tomato, the overexpression of *GhHSP24.7*, which encodes a small mitochondrial HSP, accelerates seed germination via temperature-dependent generation of reactive oxygen species (Ma et al., 2019). In *Arabidopsis*, mutation of the *AtDjA3* gene (which encodes the J protein, also referred to as HSP40 or DnaJ protein) reduces the germination of seeds when treated with ABA and increases the expression of *ABA-INSENSITIVE 3* (*ABI3*), which is a negative regulator of germination (Parcy et al., 1994; Salas-Muñoz et al., 2016). In rice, overexpression of *OsHSP18.2* improves seed germination, and a motif responsible for ABA-inducible expression is present in its promoter (Kaur et al., 2015).

MicroRNAs (miRNAs) regulate the expression of target genes by binding to and cleaving their complementary sequences or inhibiting their translation (Vaucheret, 2006; Axtell, 2013). Furthermore, the biosynthesis and function of miRNAs are regulated by plant hormones and abiotic stress in plants (Reyes and Chua, 2007; Khraiwesh et al., 2012; Sanan-Mishra et al., 2013). However, little is known about miRNAs that regulate PHS, depending on the seed maturation stage.

This study aimed to identify candidate genes and miRNAs that determine the PHS rate in rice.

## Materials and Methods

### Plant Materials and PHS Assay

Two rice (*Oryza sativa* L. ssp. *japonica*) accessions, Gopum (PHS susceptible) and Jowoon (PHS resistant), were used in this study. Both accessions were bred in an experimental field of Seoul National University, Suwon, Republic of Korea. Seeds and panicles of Gopum and Jowoon were harvested at 30, 45, and 60 days after heading (DAH). Seeds were sampled by dividing them into embryo and endosperm. To perform the PHS assay, three freshly harvested panicles were incubated at 25°C under 100% relative humidity for 7 days. The number of germinated seeds in each panicle was recorded and expressed as a percentage of the total number of seeds per panicle (Lee et al., 2017). Three replicates were used at each time point. Statistical analyses were performed using the Student’s *t-*test (**^∗^***P* < 0.05; **^∗∗^***P* < 0.01; **^∗∗∗^***P* < 0.001).

### Total RNA Extraction and Construction of mRNA and Small RNA Libraries

In total, 10 embryos and endosperms were sampled from one panicle of each plant. Total RNA was extracted from 100 embryos and endosperms, each obtained from 10 plants at three time points (30, 45, and 60 DAH). Briefly, each sample was homogenized in liquid nitrogen using a pestle and mortar and total RNA was then extracted using TRIzol Reagent (Invitrogen, United States), as described previously (Wang et al., 2012). The mRNA libraries were constructed from 2 μg of total RNA using SENSE mRNA-Seq Library Prep Kit V2 for Illumina platforms (LEXOGEN, Austria), based on the manufacturer’s instructions. Small RNAs (18–30 nt) were isolated from 20 μg of total RNA using Urea-PAGE gel, and then small RNA libraries were constructed using Small RNA-Seq Library Prep Kit for Illumina Platforms (LEXOGEN, Austria), based on the manufacturer’s instructions. Four replicates of the mRNA and small RNA samples were prepared.

### RNA-Seq Analysis

RNA-seq was performed using the Illumina HiSeq 2500 platform to generate 101-bp paired-end reads. Raw sequences were filtered and trimmed using Trimmomatic v0.3.6. The clean reads were mapped onto the International Rice Genome Sequencing Project (IRGSP) 1.0 reference genome using HISAT2 v2.1.0 with default parameters. The mapped reads were transformed into BAM files using samtools v1.8. The number of mapped reads was quantified using the featureCounts function in Rsubread v2.4.3. To estimate gene expression levels, the mapped read counts were transformed into counts per million (CPM). Subsequently, the CPM values were normalized using the trimmed mean of M-values. The log-transformed CPM values were then used to generate heatmaps with the pheatmap package v1.0.12 in R. Differentially expressed genes (DEGs) with fold-change (FC) ≥ 2 and false discovery rate (FDR) ≤ 0.05 were identified using the edgeR package v3.32.1 by comparing the two rice accessions at each time point.

### Gene Ontology Enrichment Analysis

Gene ontology (GO) enrichment analysis was performed to determine the biological roles of the selected genes (Moon et al., 2020). Significantly enriched GO terms were selected with fold enrichment > 2 and FDR < 0.05 using the GO Resource web server.^1^

### MapMan Analysis

The MapMan software v3.6.0 was used to map the transcriptome data and define functional categories for identifying significantly overrepresented functional genes (Zhang et al., 2020). A dataset containing the IDs of DEGs was constructed with two overviews: regulation and cellular response.

### Validation of RNA-Seq Data by Quantitative Real-Time PCR

To validate the RNA-seq data, cDNA was synthesized from each RNA sample using SuperScript III Reverse Transcriptase (Invitrogen, United States), based on the manufacturer’s instructions. Then, quantitative real-time PCR (qRT-PCR) was performed using Light Cycler 480 SYBR Green I Master (Roche, United States) with SYBR Green detection and gene-specific primers. The Ct values for genes were obtained using Os03g0718100 (*OsACT1*) as a control, and relative expression values were calculated using the ΔΔCt method. Gene-specific primers were designed using NCBI primer BLAST.^2^ The primer sequences of the candidate genes are listed in Supplementary Table 15. Statistical analyses were performed using the Student’s *t*-test (**^∗^***P* < 0.05; **^∗∗^***P* < 0.01; **^∗∗∗^***P* < 0.001).

### Small RNA-Seq Data Processing and Differential Expression Analysis of miRNAs

The removal of adaptor sequences and selection of 15–26 nt small RNAs were performed using Cutadapt (v3.4). To obtain high-quality small RNA reads, reads with Phred quality score < 25 were removed using Trimmomatic. The clean reads were then aligned on RNACentral (v.17.0). *Oryza* sp.-specific non-coding RNA sequences (mostly structural RNAs, including rRNAs, tRNAs, snRNAs, and snoRNAs) were filtered, and the remaining reads were mapped onto the IRGSP-1.0 rice reference genome sequence using bowtie (v1.2.2). To annotate mature rice miRNAs, the IRGSP-mapped small RNA reads were aligned against the mature rice miRNA sequences registered in miRBase (v22) and PmiREN. The filtered read counts were visualized by generating a heatmap using the pheatmap package v1.0.12 in R. Differential expression analysis of miRNAs was performed using edgeR, and miRNAs with log_2_FC > 1 and FDR < 0.05 were selected as differentially expressed miRNAs (DEMs).

## Results

### Difference in PHS Rates Between Gopum and Jowoon

**FIGURE 1:**
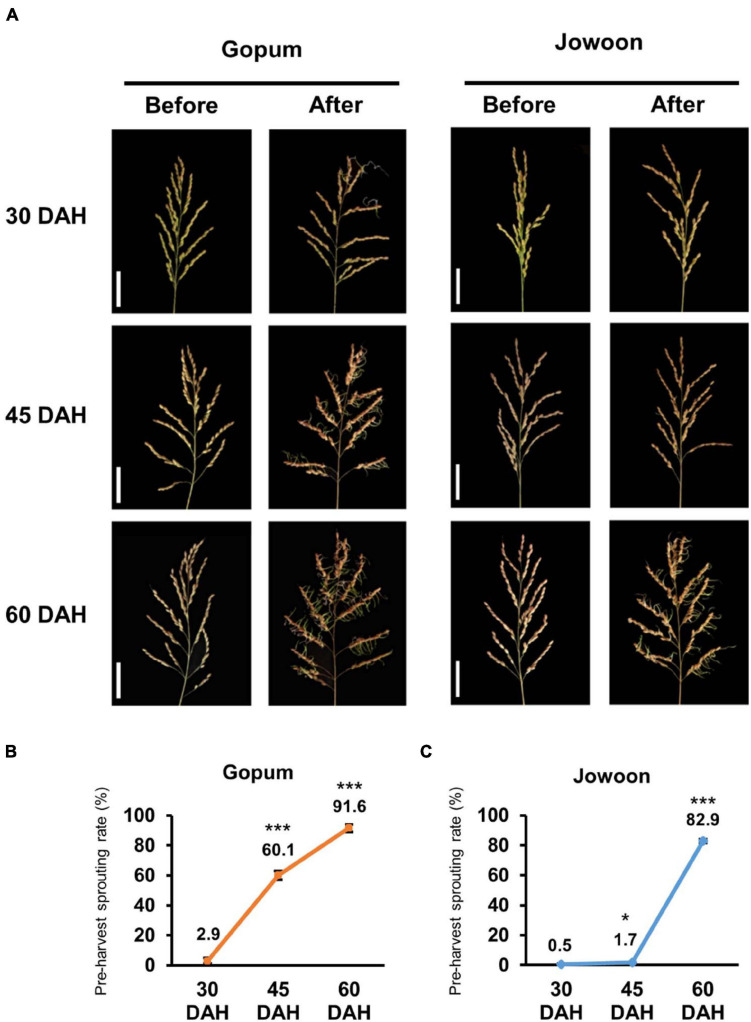
Pre-harvest sprouting (PHS) assay in Gopum and Jowoon. **(A)** PHS phenotypes of Gopum and Jowoon at 30, 45, and 60 days after heading (DAH). The phenotypes before PHS assay (before) and after PHS assay (after) are shown. Scale bar = 5 cm. **(B,C)** PHS rate of Gopum **(B)** and Jowoon **(C)** at 30, 45, and 60 DAH (*N* = 3). Significance is determined by Student’s *t-*test, *P < 0.05 and ***P < 0.001.

We defined three seed maturation stages in this study: early (30 DAH), mid (45 DAH), and late (60 DAH) stages. The PHS rate of Gopum (PHS susceptible accession) was higher than that of Jowoon (PHS resistant accession) at every stage (Figure 1A). In particular, the PHS rate of Gopum was significantly higher than that of Jowoon at 45 DAH by approximately 35-fold (Figures 1B,C). Therefore, we performed RNA-seq and RNAome analyses to identify genes and miRNAs responsible for the difference in the PHS rate between Gopum and Jowoon.

### Identification of DEGs in the Embryo and Endosperm of Gopum and Jowoon

**FIGURE 2:**
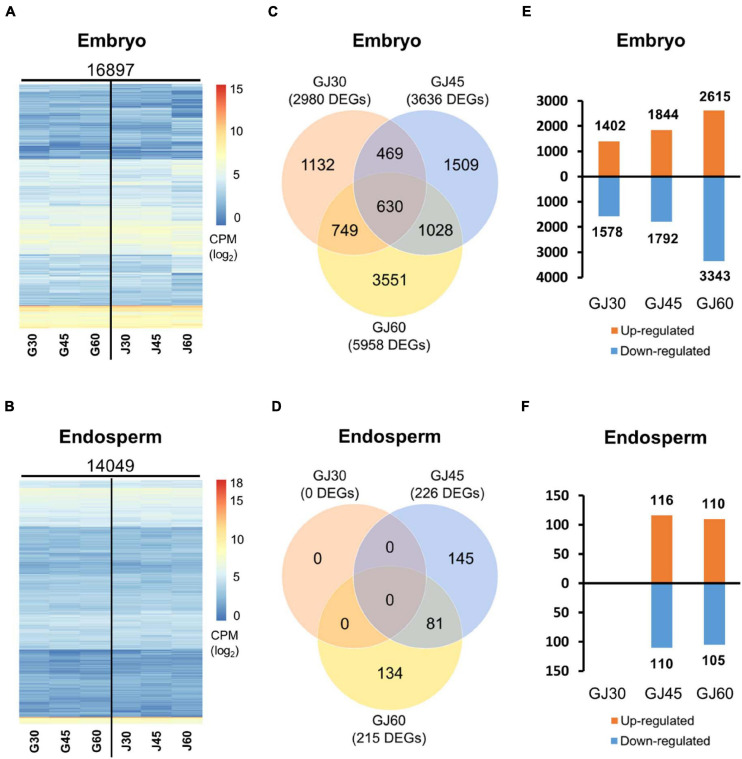
Identification of differentially expressed genes (DEGs) based on the RNA-seq data of the embryo and endosperm of Gopum and Jowoon. **(A,B)** Heatmaps of expressed genes in the embryo **(A)** and endosperm **(B)** of Gopum and Jowoon, respectively. Expressed genes, mapped onto the International Rice Genome Sequencing Project (IRGSP) rice reference genome sequence, are shown as log-transformed counts per million (CPM) values. G30, G45, G60, J30, J45, and J60 indicate Gopum and Jowoon samples at 30, 45, and 60 DAH, respectively. **(C,D)** Comparison of Gopum and Jowoon based on the number of DEGs in the embryo **(C)** and endosperm **(D)**. GJ30, GJ45, and GJ60 indicate the comparisons between Gopum and Jowoon at 30, 45, and 60 DAH, respectively. **(E,F)** Numbers of genes up- or down-regulated in Jowoon compared with those in Gopum in the embryo **(E)** and endosperm **(F)** of GJ30, GJ45, and GJ60.

To understand the transcriptional changes in PHS-related candidate genes at different seed maturation stages in Gopum and Jowoon, we performed RNA-seq analysis of the embryo and endosperm at 30, 45, and 60 DAH. After read filtering and trimming, the clean reads were mapped onto the IRGSP 1.0 rice reference genome sequence. The results showed that 16,897 and 14,049 genes were expressed in the embryo and endosperm samples, respectively. The log-transformed CPM values of each gene are depicted as a heatmap in Figures 2A,B.

To identify PHS-related candidate genes, gene expression was compared between Gopum and Jowoon samples at 30, 45, and 60 DAH (hereafter referred to as GJ30, GJ45, and GJ60, respectively). In the embryo samples, 2,980, 3,636, and 5,958 DEGs were identified in the GJ30, GJ45, and GJ60, respectively (Figure 2C). In the endosperm samples, no DEG was identified in GJ30, whereas 226 and 215 DEGs were identified in GJ45 and GJ60, respectively (Figure 2D). Genes that were up- or down-regulated in Jowoon compared with those in Gopum in GJ30, GJ45, and GJ60 of embryo and endosperm are shown in Figures 2E,F. Interestingly, the endosperm had significantly less DEGs compared with the embryo. Thus, it is likely that DEGs in the embryo cause the difference in PHS rate between Gopum and Jowoon.

### Functional Annotation of DEGs

**FIGURE 3:**
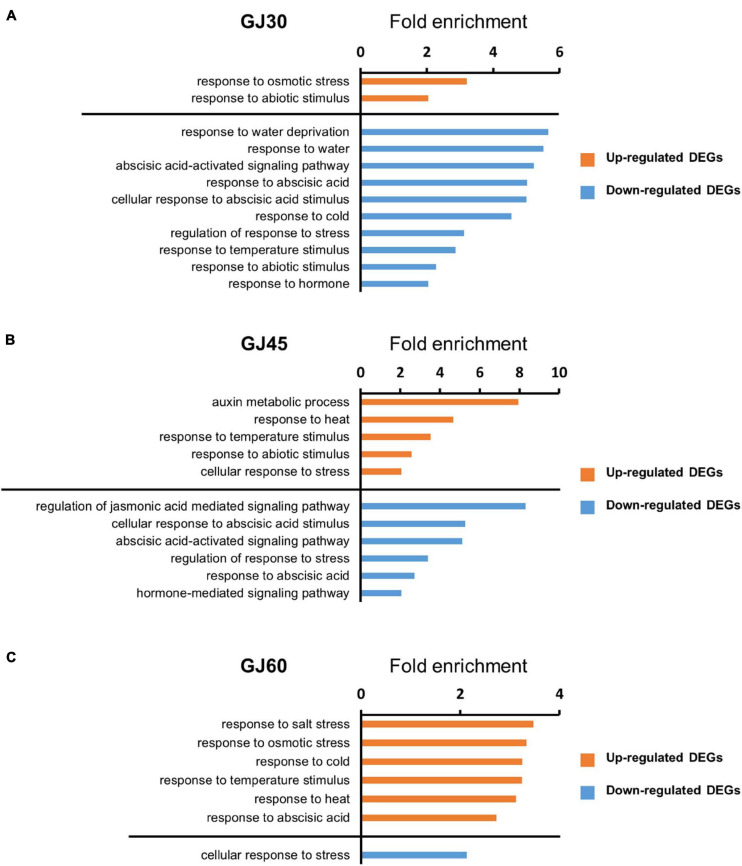
Gene ontology (GO) enrichment analysis of differentially expressed genes (DEGs) identified in the embryo in the biological process category. **(A–C)** GO terms (fold enrichment > 2 and false discovery rate < 0.05) of DEGs, which were up- or down-regulated in Jowoon compared with those in Gopum, related to hormones and abiotic stress in the embryo of GJ30 **(A)**, GJ45 **(B)**, and GJ60 **(C)**.

We performed GO enrichment analysis to predict the biological functions of DEGs in the embryo and endosperm. GO terms in the biological process category with fold enrichment > 2 and FDR < 0.05 were identified. In the embryo samples, DEGs identified in the GJ30, GJ45, and GJ60 were enriched with hormone-related GO terms, such as “response to abscisic acid,” and abiotic stress-related GO terms, such as “response to heat” (Figure 3). In endosperm samples, no significant GO term related to hormones or abiotic stress was identified (Supplementary Tables 7–9). GO term was not identified in the genes down-regulated in Jowoon, compared with those in Gopum of endosperm GJ60. All GO terms in the biological process category are listed in Supplementary Tables 1–9.

### Identification of Hormone- and Abiotic Stress-Related DEGs in the Embryo

**FIGURE 4:**
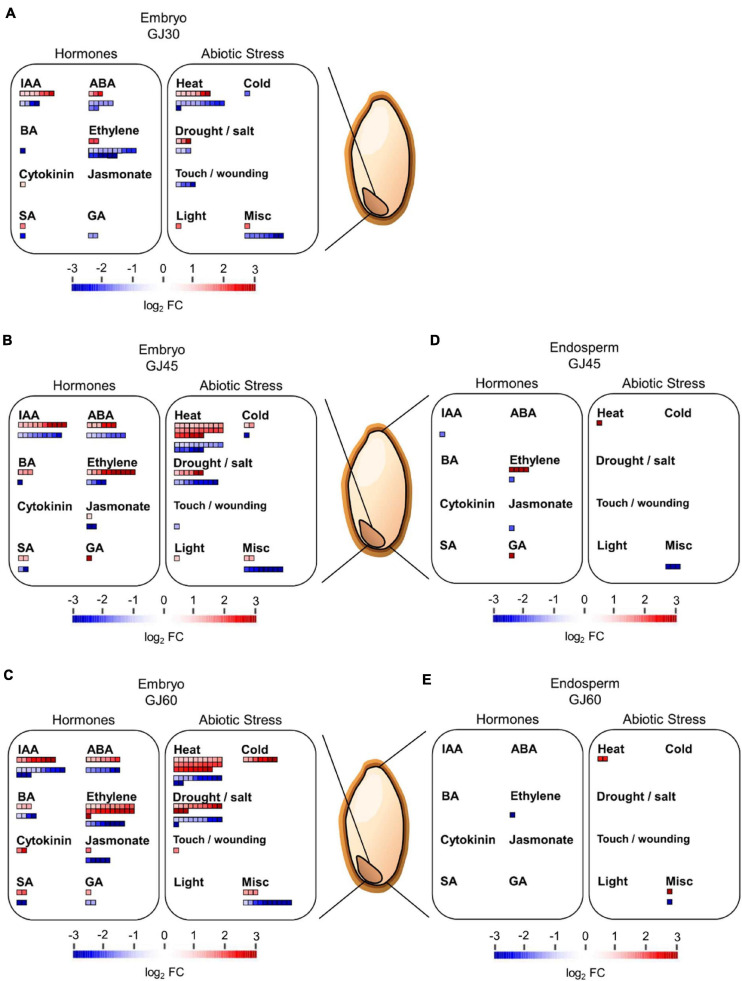
MapMan analysis of differentially expressed genes (DEGs), identified in the embryo and endosperm of Gopum and Jowoon. **(A–E)** Identification of hormone- and abiotic stress-related DEGs in the embryo of GJ30 **(A)**, GJ45 **(B)**, and GJ60 **(C)** and in endosperm of GJ45 **(D)** and GJ 60 **(E)**. The regulation overview and cellular response overview were applied, and the figures were modified from Supplementary Figures 1–4 for DEGs related to hormones and abiotic stress.

High-throughput transcriptome data can be visualized in diverse overviews using the MapMan software, based on multiple omics data of plants (Usadel et al., 2005; Jung and An, 2012). In this study, we used the MapMan software to visualize the function and expression profiles of DEGs at each seed maturation stage in the embryo and endosperm samples of Gopum and Jowoon. The DEGs identified in the embryo (GJ30, GJ45, and GJ60) and endosperm (GJ45 and GJ60) were analyzed using the regulation and cellular response overviews. The hormone-related DEGs identified in the regulation overview, showed the highest changes in indole-3-acetic acid (IAA)-, ABA-, and ethylene-related genes in the embryo of GJ30, GJ45, and GJ60. In addition, in the abiotic stress of the cellular response overview, heat stress-related genes showed the highest expression change in the embryo of GJ30, GJ45, and GJ60 (Figures 4A–C). In the endosperm, no significant changes in hormone− and abiotic stress-related genes were observed in the both GJ45 and GJ60 (Figures 4D,E). These results suggest that IAA−, ABA−, ethylene−, and heat stress-related genes with differential expression between the embryos of Gopum and Jowoon are responsible for the difference in the PHS rate between the two accessions. The regulation and cellular response overviews of all GJ30, GJ45, and GJ60 of embryo and endosperm are shown in Supplementary Figures 1–4.

### Genes Potentially Responsible for the Difference in the PHS Rate Between Gopum and Jowoon

**FIGURE 5:**
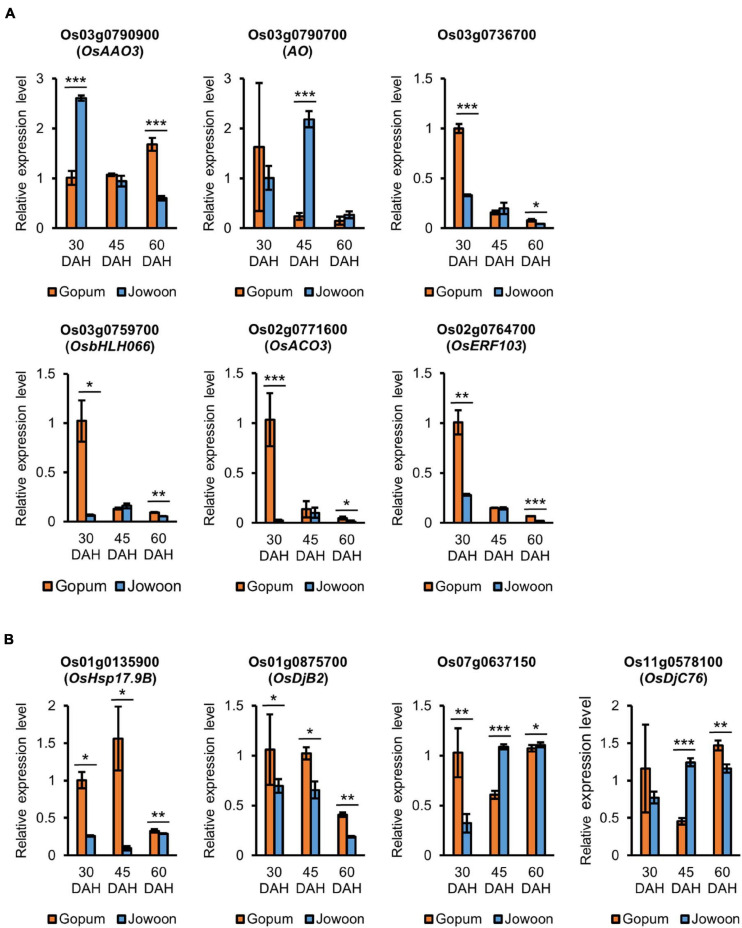
Relative expression levels of pre-harvest sprouting (PHS)-related candidate genes by quantitative real-time PCR (qRT-PCR). **(A)** Relative expression levels of abscisic acid (ABA)- and ethylene-related genes. ABA-related genes (top) and ethylene-related genes (bottom) are shown. **(B)** Relative expression levels of *heat shock protein* (*HSP*) genes. Data represent mean ± standard error of mean (SEM; *N* = 3). Significance is determined by Student’s *t-*test, *P < 0.05, **P < 0.01, and ***P < 0.001.

We found that several genes, including *AAO*, *AO*, *GRAM* domain containing gene, *bHLH*, *ACO*, *ERF*, and *HSP*, were differentially expressed between the embryos of Gopum and Jowoon at 30, 45, and 60 DAH. The review of literature, Rice Annotation Project Database (RAP-DB) and MSU Rice Genome Annotation Project, and related searches revealed several hormone and abiotic stress-related genes with known or expected functions in the embryo (Supplementary Tables 10–12). In addition, we selected PHS-related candidate genes, which have not yet been published. The expression of these genes was validated by qRT-PCR (Figure 5).

Os03g0790900 (*OsAAO3*), Os03g0790700 (*AO*), and Os03g0736700 (which contains the GRAM domain) were selected as ABA-related genes, and Os03g0759700 (*OsbHLH066*), Os02g0771600 (*OsACO3*), and Os02g0764700 (*OsERF103*) were selected as ethylene-related genes. Among these genes, Os03g0790900 (*OsAAO3*) and Os03g0790700 (*AO*) were up-regulated in Jowoon compared with those in Gopum at 30 and 45 DAH, respectively (Figure 5A). Our results suggest that Os03g0790900 (*OsAAO3*) and Os03g0790700 (*AO*) are associated with ABA and affect the low PHS rate of Jowoon at 30 and 45 DAH, respectively. In addition, Os03g0736700 (which contains the GRAM domain), Os03g0759700 (*OsbHLH066*), Os02g0771600 (*OsACO3*), and Os02g0764700 (*OsERF103*) were up-regulated in Gopum compared with those in Jowoon at 30 DAH (Figure 5A). These results suggest that the abovementioned genes are associated with ethylene and affect the higher PHS rate of Gopum compared with that of Jowoon at 45 DAH.

The selected *HSP* genes including Os01g0135900 (*OsHsp17.9B*), Os01g0875700 (*OsDjB2*), Os07g0637150 (which is similar to *DnaJ*), and Os11g0578100 (*OsDjC76*) were up-regulated in Gopum compared with those in Jowoon at 30 DAH (Figure 5B). These results suggest that the abovementioned *HSP* genes are associated with the higher PHS rate of Gopum compared with that of Jowoon at 45 DAH. In addition, the high expression of Os07g0637150 and Os11g0578100 (*OsDjC76*) at 45 DAH in Jowoon is associated with the PHS of Jowoon seeds at 60 DAH (Figure 5B).

Details of hormone and abiotic stress-related genes in GJ30, GJ45, and GJ60 of the embryo and endosperm, respectively, are summarized in Supplementary Tables 10–14. All primers used for qRT-PCR are listed in Supplementary Table 15.

### Identification of PHS-Related Candidate miRNAs and Corresponding Target Genes

**FIGURE 6:**
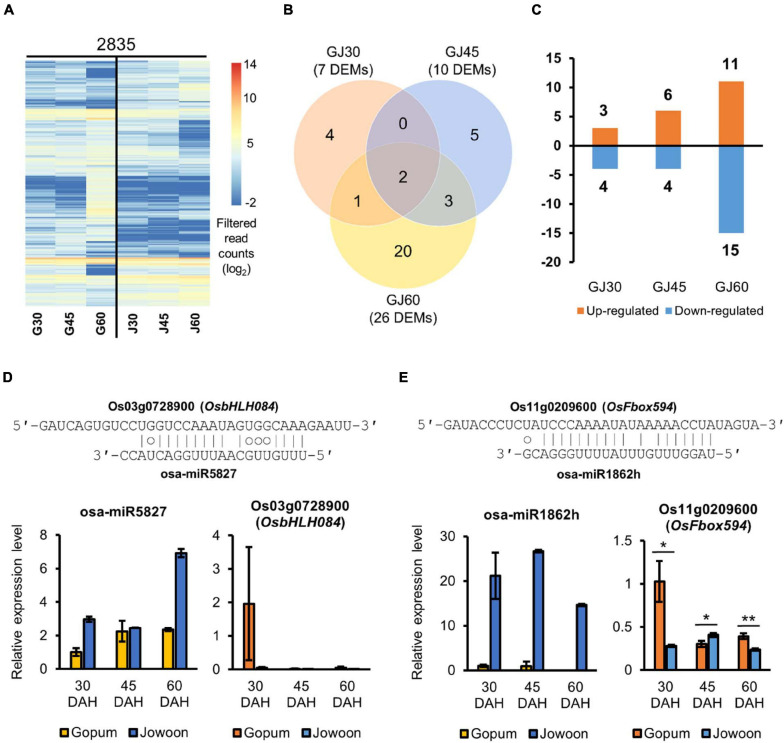
Identification and expression analysis of differentially expressed miRNAs (DEMs) and the corresponding target genes. **(A)** Heat map of the expressed miRNAs in the embryo of Gopum and Jowoon. Expressed miRNAs, aligned to miRBase and PmiREN, were shown as filtered read counts (log_2_) values. **(B)** Comparison of Gopum and Jowoon based on the number of DEMs in the embryo of GJ30, GJ45, and GJ60. **(C)** Number of miRNAs up- or down-regulated in Jowoon compared with those in Gopum in the embryo of GJ30, GJ45, and GJ60. **(D,E)** Relative expression levels of miRNAs and corresponding target genes, which were predicted to affect PHS. The pairing between miRNAs and their target genes is indicated. The relative expression levels of genes were validated by qRT-PCR. Data represent mean ± SEM (*N* = 3). Significance is determined by Student’s *t-*test, *P < 0.05 and **P < 0.01.

On the basis of our transcriptome data, we speculated that the embryo plays a greater role in PHS than the endosperm. Furthermore, we predicted that miRNAs associated with PHS would be expressed in the embryo. To identify miRNAs and their corresponding target genes affecting the difference in PHS rate between Gopum and Jowoon, we performed small RNA-seq analysis of Gopum and Jowoon embryos at 30, 45, and 60 DAH. After read filtering and trimming, the clean small RNA reads were mapped onto the IRGSP 1.0 rice reference genome sequence. The IRGSP-mapped small RNA reads were aligned against the mature rice miRNA sequences in miRBase and PmiREN. Consequently, 2,835 miRNAs were identified in the embryo samples. The heatmap of filtered read counts of miRNAs is shown in Figure 6A. Next, we performed DEM analysis (log_2_FC > 1; FDR < 0.05) at each stage to identify PHS-related candidate miRNAs. In total 7, 10, and 26 DEMs were identified in the GJ30, GJ45, and GJ60, respectively (Figure 6B). The up- and down-regulated miRNAs in Jowoon compared with those in Gopum in GJ30, GJ45, and GJ60 are shown in Figure 6C.

The target genes of DEMs were identified using miRNA target gene prediction. We considered the anti-correlation in expression level between DEMs and the corresponding target genes and validated the expression of target genes by qRT-PCR. Subsequently, two miRNAs and two target genes, expected to be associated with PHS, were selected (Figures 6D,E). The osa-miR5827 and osa-miR1862h were expressed at higher levels in Jowoon than in Gopum at all stages, whereas the expression of the corresponding target genes, Os03g0728900 (*OsbHLH084*) and Os11g0209600 (*OsFbox594*), respectively, was lower in Jowoon than in Gopum at 30 DAH (Figures 6D,E). These results suggest that Os03g0728900 (*OsbHLH084*) and Os11g0209600 (*OsFbox594*) are associated with the higher PHS rate of Gopum compared with that of Jowoon at 45 DAH. In addition, the high expression of osa-miR5827 and osa-miR1862h in Jowoon might be associated with the low PHS rate of Jowoon, especially the case with osa-miR1862h.

The DEMs and their corresponding target genes are summarized in Supplementary Tables 16–18.

## Discussion

Rice is one of the most important crops in the world. However, the reduction in rice grain quality and yield due to PHS results in a significant economic loss (Gubler et al., 2005; Wei et al., 2013). Therefore, to overcome PHS, it is necessary to understand the molecular mechanisms underlying seed dormancy and maturation, as well as genes and miRNAs involved in PHS. In this study, we performed RNA-seq and small RNA-seq analyses of the embryo and endosperm tissues of the PHS susceptible accession Gopum and PHS resistant accession Jowoon at three seed maturation stages: 30, 45, and 60 DAH.

Our RNA-seq data revealed the expression of *OsSdr4*, *OsPYL/RCAR5*, and *OsDOG1L-3*, which are well known to negatively control seed germination (Sugimoto et al., 2010; Graeber et al., 2012; Kim et al., 2012; Wang et al., 2020). The expression of Os07g0585700 (*OsSdr4*) was higher in Jowoon than in Gopum at 60 DAH. In addition, the expression of Os05g0213500 (*OsPYL/RCAR5*) and Os01g0306400 (*OsDOG1L-3*) was overall higher in Jowoon than in Gopum; however, no significant expression changes were noted between Gopum and Jowoon based on the seed maturation stages (Supplementary Figure 5).

**FIGURE 7:**
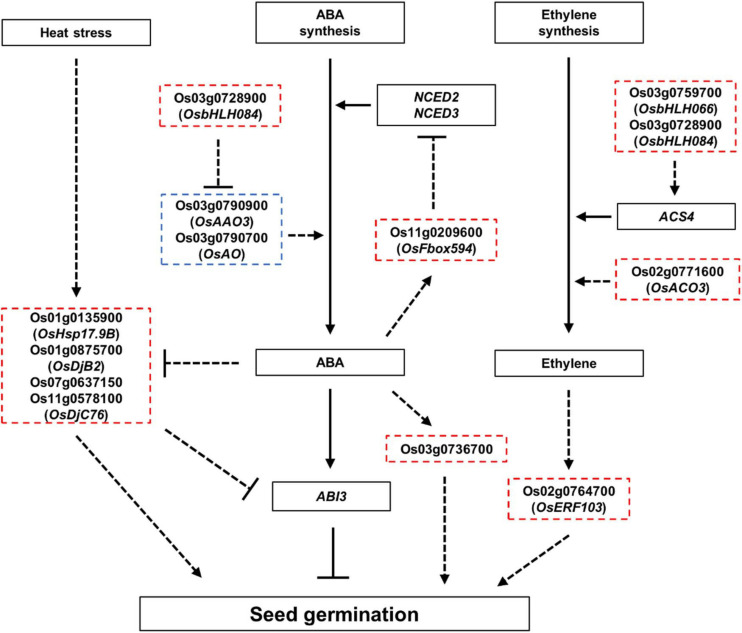
Proposed model showing the role of ABA, ethylene, and HSPs in seed germination. Red and blue dashed boxes indicate genes expected to positively and negatively regulate the seed germination, respectively. Arrows indicate positive regulation, and bars indicate negative regulation. Bold lines indicate gene functions supported by strong evidence, and dashed lines indicate gene function with limited evidence in seed germination, which requires further investigation.

The results of the GO enrichment and MapMan analyses revealed that hormone- and heat stress-related GO terms and gene expression differences between Gopum and Jowoon were significantly observed in the embryo and rarely in the endosperm (Figures 3, 4 and Supplementary Tables 7–9). These results suggest that the expression of hormone- and heat stress-related genes in embryo plays a crucial role in PHS. Thus, using the embryo data, we identified genes that are expected to affect PHS. We identified three ABA- and three ethylene-related genes. *AAO* is involved in the last step of ABA biosynthesis. Previously, *aao3* and *ao3* mutants showed reduced seed dormancy and ABA levels (González-Guzmán et al., 2004; Shi et al., 2021). The expression of Os03g0790900 (*OsAAO3*) and Os03g0790700 (*AO*) was higher in Jowoon than in Gopum at 30 and 45 DAH, respectively (Figure 5A). It is possible that the high expression level of Os03g0790900 (*OsAAO3*) and Os03g0790700 (*AO*) is associated with high ABA levels in Jowoon seeds at 30 and 45 DAH, respectively, resulting in low PHS rate (Figure 7). GEM, which contains a GRAM domain, is involved in ABA signaling and is activated by ABA, and the *Arabidopsis gem-1* mutant shows increased seed dormancy (Mauri et al., 2016). We identified Os03g0736700, which contains a GRAM domain, as a DEG whose expression level was higher in Gopum than in Jowoon at 30 DAH (Figure 5A). We predict that Os03g0736700 responds to ABA and affects the high PHS rate of Gopum after 30 DAH (Figure 7).

Ethylene promotes seed germination (Arc et al., 2013; Corbineau et al., 2014). ACS synthesizes the metabolic precursor of ethylene, ACC, which is oxidized by ACO to form ethylene (Zarembinski and Theologis, 1994; Wang et al., 2002). In *Arabidopsis*, *PIF5* overexpression lines showed high *ACS4* expression and increased ethylene level (Khanna et al., 2007). In addition, the *aco2* mutant showed reduced seed germination (Linkies et al., 2009). The *aterf7* mutant showed delayed seed germination, whereas tomato *SlERF2* overexpression lines showed early seed germination than the wild type (Song et al., 2005; Pirrello et al., 2006). Os03g0759700 (*OsbHLH066*, which encodes a bHLH transcription factor), Os02g0771600 (*OsACO3*), and Os02g0764700 (*OsERF103*) showed higher expression in Gopum than in Jowoon at 30 DAH (Figure 5A). Therefore, we speculate that these three genes, involved in ethylene biosynthesis and response, contribute to the high PHS rate of Gopum at 45 DAH (Figure 7).

HSPs, associated with ABA, control seed germination. In *Arabidopsis* and tomato, the overexpression of *GhHSP24.7* accelerated seed germination (Ma et al., 2019). In addition, the *Atdja3* mutant showed reduced seed germination, when treated with ABA, and higher expression of *ABI3* than the wild type (Salas-Muñoz et al., 2016). In rice, an ABA-inducible motif was identified in the *OsHSP18.2* promoter region, and *OsHSP18.2* overexpression lines showed enhanced seed germination (Kaur et al., 2015). In the present study, Os01g0135900 (*OsHsp17.9B*) and Os01g0875700 (*OsDjB2*) showed higher expression in Gopum than in Jowoon at all stages and are, therefore, expected to contribute to the overall high PHS rate of Gopum (Figure 5B). In addition, Os07g0637150 (similar to *DnaJ*) showed higher expression in Gopum at 30 DAH, whereas Os11g0578100 (*OsDjC76*) showed higher expression in Jowoon at 45 DAH (Figure 5B). We speculate that these genes are associated with the high PHS rate of Gopum at 45 DAH and that of Jowoon at 60 DAH. On the basis of previous studies (Kaur et al., 2015; Salas-Muñoz et al., 2016), the *HSP* genes, which showed low expression in Jowoon, may be affected by ABA synthesized from the high expression of Os03g0790900 (*AAO3*) and Os03g0790700 (*AO*). These *HSP* genes may affect the low PHS rate in Jowoon by enhancing the expression of *ABI3*, which is a negative regulator of seed germination (Figures 5, 7).

We speculate that the difference in the PHS rates of Gopum and Jowoon is caused by the difference in gene expression in the embryo. Little is known about the role of miRNAs in PHS. Therefore, we performed small RNA-seq analysis of the embryo. Using target prediction, we identified F-box and bHLH-related genes as the targets of DEMs from our small RNAome data (Supplementary Tables 16, 17). We selected osa-miR5827, osa-miR1862h, Os03g0728900 (*OsbHLH084*), and Os11g0209600 (*OsFbox594*), considering the anti-correlation between miRNA and the corresponding target genes (Figure 6). We speculate that the higher expression of osa-miR5827 and osa-miR1862h in Jowoon and that of Os03g0728900 (*OsbHLH084*) and Os11g0209600 (*OsFbox594*) in Gopum at 30 DAH affects the PHS phenotype of the two accessions. In previous studies, *PIF5* and the *OsbHLH035* were reported to be associated with ethylene biosynthesis (by affecting *ACS4* expression) and ABA biosynthesis (by affecting *AAO3* expression), respectively (Khanna et al., 2007; Chen et al., 2018). *OsbHLH084*, which was identified in this study, is expected to be involved in ethylene biosynthesis by affecting the expression of *ACS4* or in ABA biosynthesis by lowering the expression of Os03g0790900 (*OsAAO3*) and Os03g0790700 (*AO*) in Gopum compared with their expression in Jowoon at 30 and 45 DAH, respectively, leading to the high PHS rate of Gopum after 30 DAH (Figure 7). In addition, on the basis of a previous study (Song et al., 2012), it can be expected that Os11g0209600 (*OsFbox594*) represses the ABA biosynthesis gene, NCED, and affects the high PHS rate of Gopum after 30 DAH (Figure 7).

## Conclusion

In conclusion, by comparing the PHS susceptible rice Gopum with the PHS resistant rice Jowoon, we identified eight hormone-related genes, four HSP-related genes, and two miRNAs as the potential regulators of the difference in PHS between Gopum and Jowoon. In addition, we demonstrated a bioinformatics pipeline that can be used to identify candidate genes and miRNAs involved in PHS; this involves RNA-seq and small RNA-seq analyses and the MapMan analysis of the transcriptome data. A comprehensive review of the previous studies and the results of the present study suggest that interaction between ABA and ABA-related genes and between ABA and HSPs contributes to seed dormancy during seed maturation. On the basis of these results, we propose a model in which seed germination is affected by ABA, ethylene, and HSPs (Figure 7). Additional studies, such as the mutagenesis of hormone-related and *HSP* genes identified in this study and the measurement of ABA and ethylene contents, are needed to elucidate the interaction between ABA and ABA-related genes, ethylene and ethylene-related genes, and ABA and HSPs. Overall, this study provides big data on the expression dynamics of hormone- and abiotic stress-related genes and miRNAs, which can be utilized in various ways to manipulate seed maturation and PHS.

## Data Availability Statement

The original contributions presented in the study are publicly available. The datasets generated and analyzed for this study can be found in the NCBI SRA repository, https://www.ncbi.nlm.nih.gov/sra/, with the GEO accession no. GSE174017 and no. GSE175561.

## Author Contributions

CS conceived the research and designed the experiments. MP, DL, J-SJ, and CS planned and performed the field experiments, sampling, and PHS assay. WC, S-YS, HM, Y-SG, and K-HJ performed computational analysis. CS and MP prepared the manuscript. All authors read and approved the manuscript.

## Conflict of Interest

The authors declare that the research was conducted in the absence of any commercial or financial relationships that could be construed as a potential conflict of interest.

## Publisher’s Note

All claims expressed in this article are solely those of the authors and do not necessarily represent those of their affiliated organizations, or those of the publisher, the editors and the reviewers. Any product that may be evaluated in this article, or claim that may be made by its manufacturer, is not guaranteed or endorsed by the publisher.

## Footnotes

1
http://geneontology.org

2
http://www.ncbi.nlm.nih.gov/tools/primer-blast/
