# Supplementary material for: Identification of Genes and MicroRNAs Affecting Pre-harvest Sprouting in Rice (Oryza sativa L.) by Transcriptome and Small RNAome Analyses
Source: Front Plant Sci. 2021 Aug 6;12:727302. doi: 10.3389/fpls.2021.727302 (PMC8377729; doi:10.3389/fpls.2021.727302)
Supplement: Supplementary file 1 [file Data_Sheet_1.zip › Supplementary Figures S1 - S5.pdf]

# *Supplementary Material*

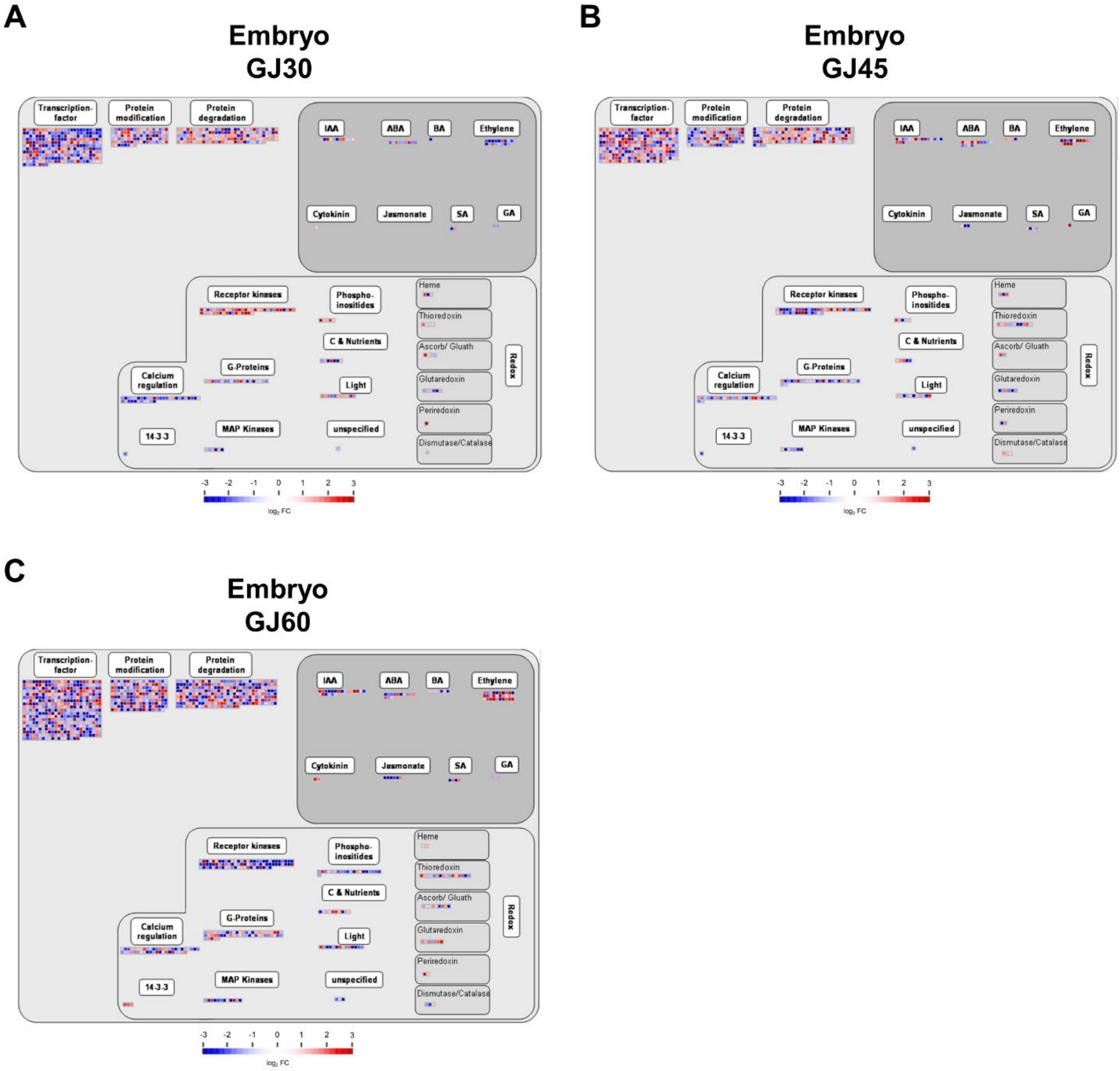


**Supplementary Figure S1.** Regulation overview of embryo using MapMan. **(A-C)** Regulation overview of embryo GJ30 (A), GJ45 (B) and GJ60 (C).

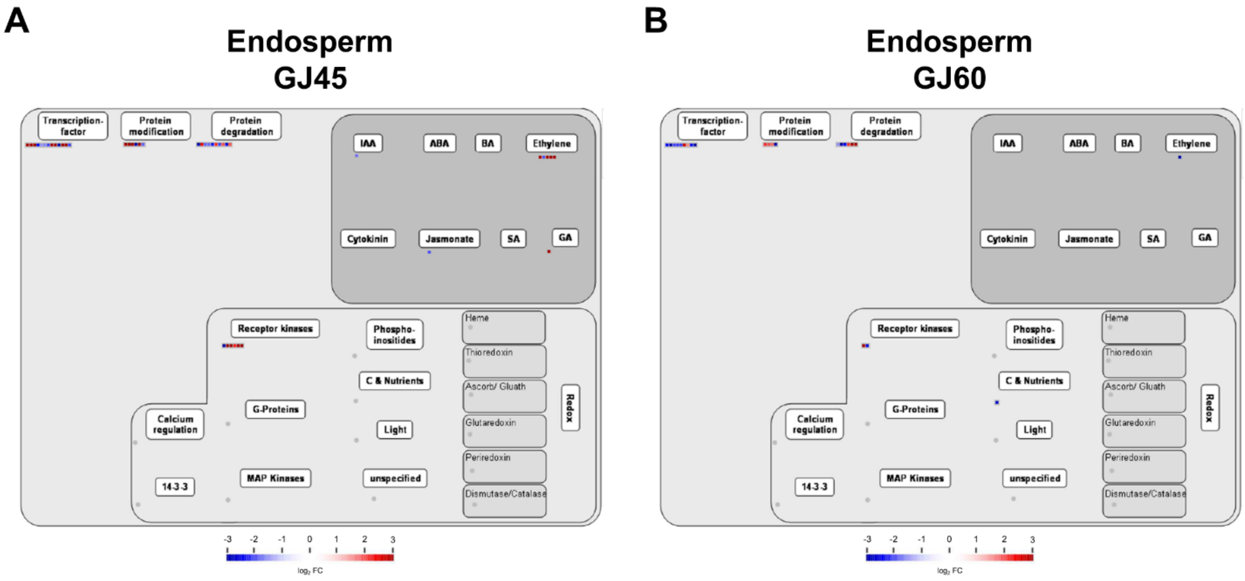


**Supplementary Figure S2.** Regulation overview of endosperm using MapMan. **(A-B)** Regulation overview of endosperm GJ45 (A), and GJ60 (B).

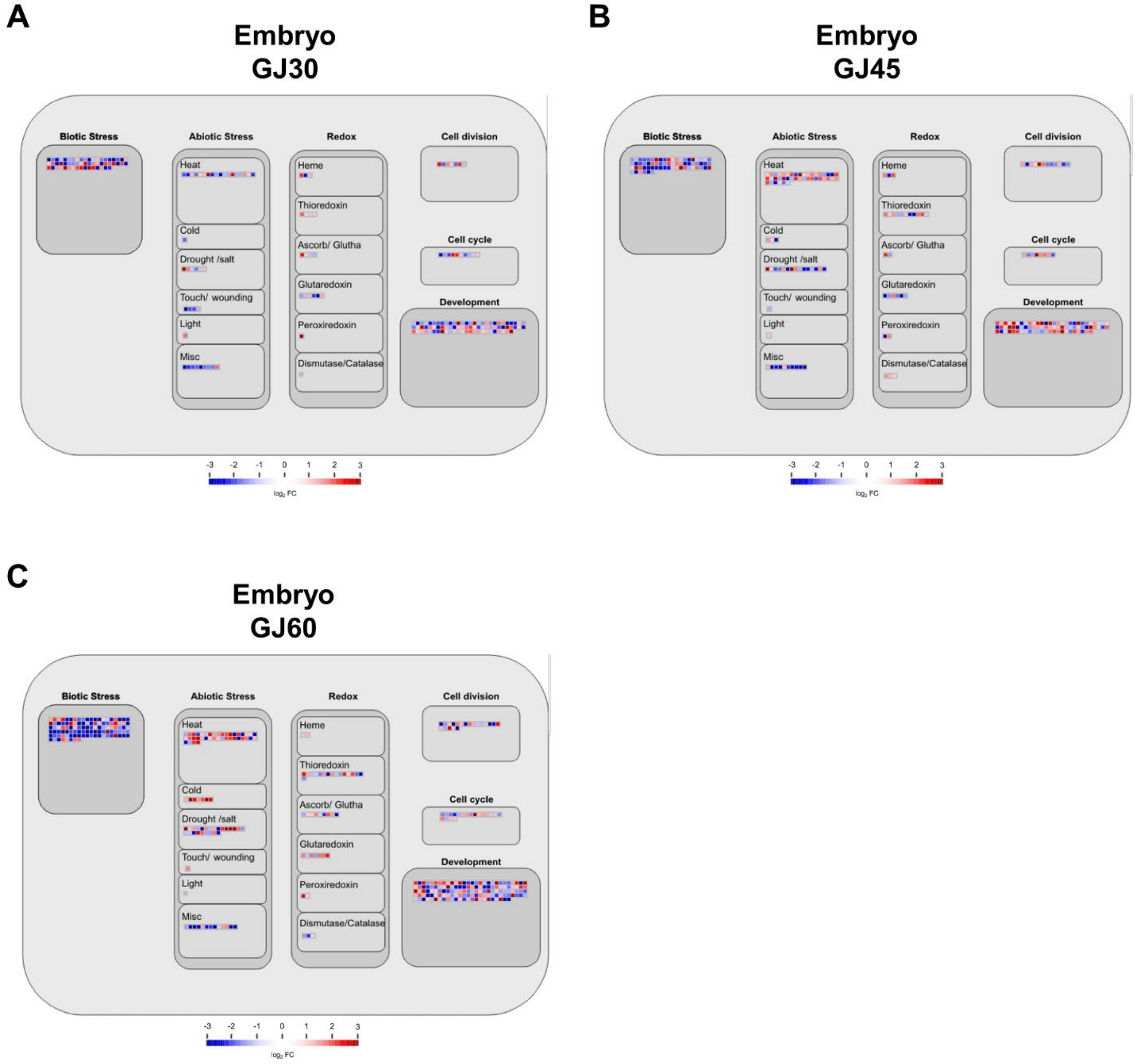


**Supplementary Figure S3.** Cellular response overview of embryo using MapMan. **(A-C)** Cellular response overview of embryo GJ30 (A), GJ45 (B), and GJ60 (C).

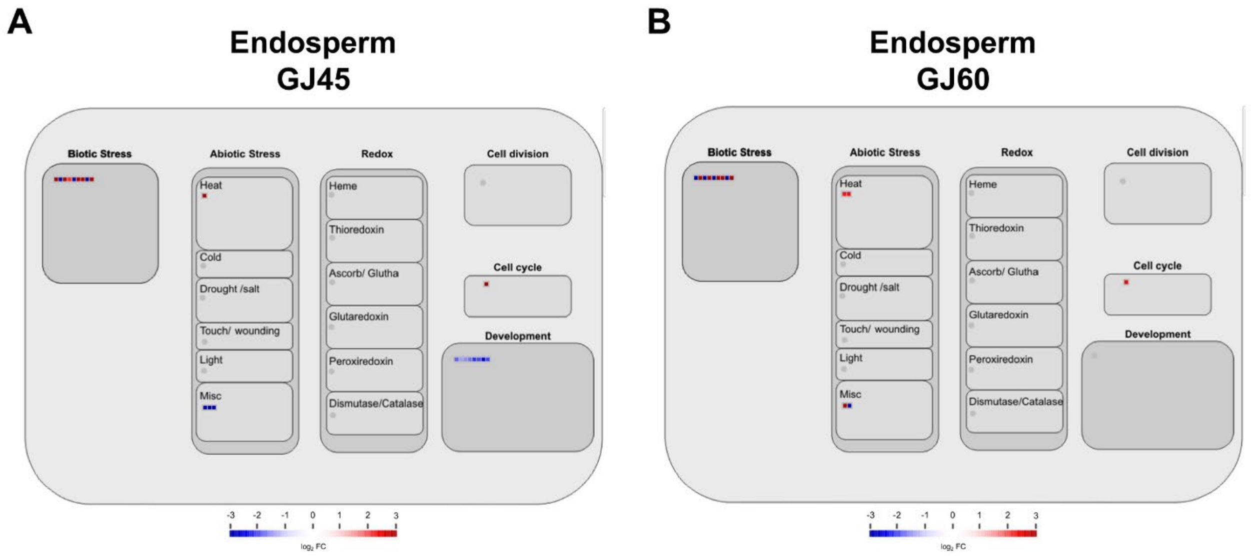


**Supplementary Figure S4.** Cellular response overview of endosperm using MapMan. **(A-B)** Cellular response overview of embryo GJ45 (A), and GJ60 (B).

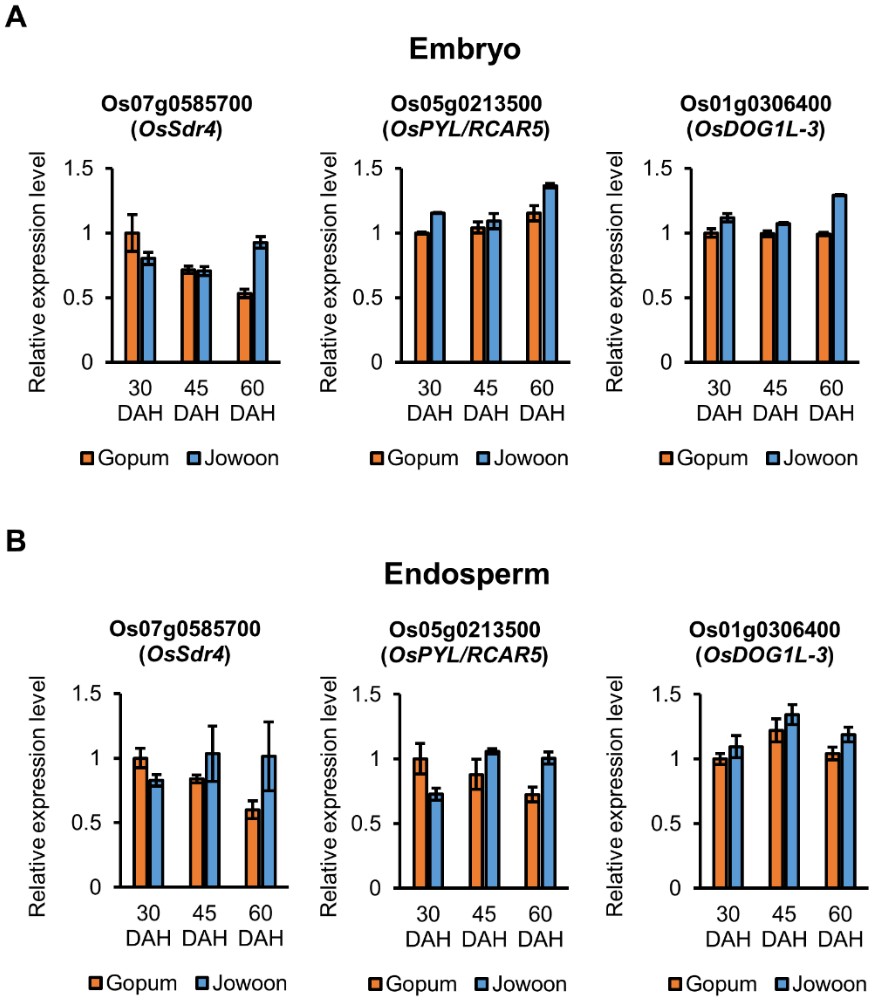


**Supplementary Figure S5.** Relative expression levels of *OsSdr4*, *OsPYL/RCAR5*, and *OsDOG1L-3* in embryo **(A)** and endosperm **(B)** using log-transformed counts per million (CPM) values in RNA-seq data. Data represent mean ± standard error of mean (SEM).
