# Supplementary material for: Identification of Genes and MicroRNAs Affecting Pre-harvest Sprouting in Rice (Oryza sativa L.) by Transcriptome and Small RNAome Analyses
Source: Front Plant Sci. 2021 Aug 6;12:727302. doi: 10.3389/fpls.2021.727302 (PMC8377729; doi:10.3389/fpls.2021.727302)
Supplement: Supplementary file 1 [file Data_Sheet_1.zip › Supplementary Tables S1 - S9.PDF]

Supplementary Table S1. GO terms belonging to the "biological process" category of up-regulated genes in the embryo GJ30 (fold enrichment > 2, FDR < 0.05)

GO term	Fold enrichment	FDR
cellular response to amino acid stimulus (GO:0071230)	24.42	2.85E-02
response to amino acid (GO:0043200)	19.53	4.04E-02
cellular response to organonitrogen compound (GO:0071417)	19.53	4.01E-02
ribosomal large subunit export from nucleus (GO:0000055)	14.47	1.74E-02
COPII-coated vesicle cargo loading (GO:0090110)	13.57	5.24E-03
methylguanosine-cap decapping (GO:0110156)	12.52	6.73E-03
deadenylation-dependent decapping of nuclear-transcribed mRNA (GO:0000290)	11.84	2.86E-02
mRNA destabilization (GO:0061157)	11.84	2.84E-02
P-body assembly (GO:0033962)	11.84	2.82E-02
RNA destabilization (GO:0050779)	11.84	2.80E-02
vesicle cargo loading (GO:0035459)	11.63	8.42E-03
RNA decapping (GO:0110154)	11.63	8.34E-03
protein import into nucleus (GO:0006606)	11.1	1.60E-08
positive regulation of mRNA catabolic process (GO:0061014)	10.85	3.43E-02
import into nucleus (GO:0051170)	10.85	2.03E-08
protein localization to nucleus (GO:0034504)	10.39	3.20E-08
COPII-coated vesicle budding (GO:0090114)	10.17	1.31E-02
ribosomal subunit export from nucleus (GO:0000054)	8.14	2.70E-02
rRNA-containing ribonucleoprotein complex export from nucleus (GO:0071428)	8.14	2.68E-02
nuclear transport (GO:0051169)	8.14	1.04E-11
ribosome localization (GO:0033750)	8.14	2.66E-02
nucleocytoplasmic transport (GO:0006913)	8.14	9.89E-12
SCF-dependent proteasomal ubiquitin-dependent protein catabolic process (GO:0031146)	6.87	5.68E-08
nuclear export (GO:0051168)	6.4	1.07E-04
regulation of catabolic process (GO:0009894)	6.3	4.91E-07
RNA export from nucleus (GO:0006405)	6.17	3.46E-04
regulation of cellular catabolic process (GO:0031329)	6.05	7.32E-05
positive regulation of catabolic process (GO:0009896)	6.05	7.22E-05
protein import (GO:0017038)	5.62	9.44E-07
establishment of RNA localization (GO:0051236)	5.56	7.26E-05
RNA transport (GO:0050658)	5.56	7.15E-05
nucleic acid transport (GO:0050657)	5.56	7.05E-05
protein export from nucleus (GO:0006611)	5.32	1.14E-02
positive regulation of protein catabolic process (GO:0045732)	5.05	7.32E-03
RNA localization (GO:0006403)	5.01	1.84E-04
ribonucleoprotein complex export from nucleus (GO:0071426)	4.95	3.16E-02
ribonucleoprotein complex localization (GO:0071166)	4.95	3.14E-02
positive regulation of cellular catabolic process (GO:0031331)	4.91	1.74E-02
nuclear-transcribed mRNA catabolic process, deadenylation-dependent decay (GO:0000288)	4.85	3.45E-02
starch metabolic process (GO:0005982)	4.75	3.73E-02
protein-containing complex localization (GO:0031503)	4.56	4.44E-02
regulation of protein catabolic process (GO:0042176)	4.44	1.55E-02
proteasomal protein catabolic process (GO:0010498)	4.4	5.26E-09
proteasome-mediated ubiquitin-dependent protein catabolic process (GO:0043161)	4.3	2.62E-08

nuclear-transcribed mRNA catabolic process (GO:0000956)	4.28	1.66E-03
response to nutrient levels (GO:0031667)	4.2	3.56E-02
protein localization to membrane (GO:0072657)	4.16	9.55E-05
mRNA catabolic process (GO:0006402)	4.15	2.15E-03
protein polyubiquitination (GO:0000209)	4.13	2.31E-02
membrane lipid biosynthetic process (GO:0046467)	3.94	9.86E-03
establishment of protein localization to organelle (GO:0072594)	3.89	3.77E-07
nucleobase-containing compound transport (GO:0015931)	3.78	2.81E-04
RNA catabolic process (GO:0006401)	3.71	3.33E-03
protein localization to organelle (GO:0033365)	3.64	4.37E-07
cellular protein localization (GO:0034613)	3.56	6.12E-14
positive regulation of protein metabolic process (GO:0051247)	3.56	4.67E-03
cellular macromolecule localization (GO:0070727)	3.5	1.13E-13
intracellular protein transport (GO:0006886)	3.46	1.01E-11
energy derivation by oxidation of organic compounds (GO:0015980)	3.44	3.86E-03
membrane lipid metabolic process (GO:0006643)	3.26	3.30E-02
ubiquitin-dependent protein catabolic process (GO:0006511)	3.21	6.41E-09
response to osmotic stress (GO:0006970)	3.2	3.65E-02
modification-dependent protein catabolic process (GO:0019941)	3.12	8.42E-09
intracellular transport (GO:0046907)	3.1	1.11E-11
modification-dependent macromolecule catabolic process (GO:0043632)	3.06	1.53E-08
protein localization (GO:0008104)	3.04	2.75E-13
establishment of localization in cell (GO:0051649)	3.01	2.00E-11
proteolysis involved in cellular protein catabolic process (GO:0051603)	2.99	6.24E-09
cellular protein catabolic process (GO:0044257)	2.98	6.44E-09
cellular localization (GO:0051641)	2.97	1.37E-12
protein transport (GO:0015031)	2.96	1.29E-11
protein catabolic process (GO:0030163)	2.95	6.28E-09
establishment of protein localization (GO:0045184)	2.94	1.66E-11
cellular macromolecule catabolic process (GO:0044265)	2.91	4.87E-11
peptide transport (GO:0015833)	2.88	3.25E-11
amide transport (GO:0042886)	2.87	2.58E-11
macromolecule localization (GO:0033036)	2.84	1.24E-13
regulation of translation (GO:0006417)	2.77	1.73E-02
nucleobase-containing compound catabolic process (GO:0034655)	2.76	3.30E-02
regulation of cellular amide metabolic process (GO:0034248)	2.71	2.07E-02
heterocycle catabolic process (GO:0046700)	2.7	2.17E-02
negative regulation of gene expression (GO:0010629)	2.69	2.24E-03
cellular nitrogen compound catabolic process (GO:0044270)	2.67	2.35E-02
posttranscriptional regulation of gene expression (GO:0010608)	2.66	6.97E-03
nitrogen compound transport (GO:0071705)	2.65	1.02E-11
macromolecule catabolic process (GO:0009057)	2.61	6.58E-10
organonitrogen compound catabolic process (GO:1901565)	2.6	3.92E-08
organic substance transport (GO:0071702)	2.5	9.52E-12
mRNA metabolic process (GO:0016071)	2.49	1.76E-04
transcription, DNA-templated (GO:0006351)	2.49	2.06E-02
RNA biosynthetic process (GO:0032774)	2.48	1.56E-02
nucleic acid-templated transcription (GO:0097659)	2.43	2.29E-02
anion transport (GO:0006820)	2.39	1.98E-11
positive regulation of metabolic process (GO:0009893)	2.31	5.94E-04
positive regulation of macromolecule metabolic process (GO:0010604)	2.3	8.10E-04
carbohydrate derivative biosynthetic process (GO:1901137)	2.27	8.51E-03

regulation of protein metabolic process (GO:0051246)	2.25	1.26E-03
ion transport (GO:0006811)	2.24	8.34E-13
mRNA processing (GO:0006397)	2.24	2.67E-02
positive regulation of nitrogen compound metabolic process (GO:0051173)	2.22	3.33E-03
organic substance catabolic process (GO:1901575)	2.18	6.92E-09
cellular catabolic process (GO:0044248)	2.15	4.97E-08
peptidyl-amino acid modification (GO:0018193)	2.12	2.72E-02
chromosome organization (GO:0051276)	2.11	1.74E-02
regulation of biological quality (GO:0065008)	2.08	5.47E-03
negative regulation of macromolecule metabolic process (GO:0010605)	2.07	6.97E-03
response to abiotic stimulus (GO:0009628)	2.04	1.56E-02
regulation of cellular protein metabolic process (GO:0032268)	2.03	1.59E-02
negative regulation of metabolic process (GO:0009892)	2.03	9.09E-03

Supplementary Table S2. GO terms belonging to the "biological process" category of down-regulated genes in the embryo GJ30 (fold enrichment > 2, FDR < 0.05)

GO term	Fold enrichment	FDR
cold acclimation (GO:0009631)	17.23	6.44E-04
endosome transport via multivesicular body sorting pathway (GO:0032509)	9.57	1.95E-04
multivesicular body sorting pathway (GO:0071985)	9.57	1.91E-04
late endosome to vacuole transport (GO:0045324)	8.45	1.40E-04
ribosomal large subunit assembly (GO:0000027)	6.38	5.61E-03
response to wounding (GO:0009611)	5.85	7.50E-04
response to water deprivation (GO:0009414)	5.66	9.53E-05
response to water (GO:0009415)	5.51	1.17E-04
abscisic acid-activated signaling pathway (GO:0009738)	5.22	8.04E-04
response to acid chemical (GO:0001101)	5.16	2.05E-04
response to abscisic acid (GO:0009737)	5.02	2.46E-07
cellular response to abscisic acid stimulus (GO:0071215)	5	1.14E-03
cellular response to alcohol (GO:0097306)	5	1.13E-03
response to alcohol (GO:0097305)	4.92	3.28E-07
endosomal transport (GO:0016197)	4.56	8.99E-03
defense response to fungus (GO:0050832)	4.56	8.89E-03
response to cold (GO:0009409)	4.55	1.21E-03
glucose metabolic process (GO:0006006)	4.51	3.24E-02
response to fungus (GO:0009620)	4.05	1.82E-02
ribosomal large subunit biogenesis (GO:0042273)	4.02	1.92E-03
regulation of defense response (GO:0031347)	3.64	3.37E-02
negative regulation of hydrolase activity (GO:0051346)	3.39	1.35E-02
response to inorganic substance (GO:0010035)	3.3	4.96E-04
response to lipid (GO:0033993)	3.28	6.73E-05
vacuolar transport (GO:0007034)	3.19	4.90E-02
regulation of response to stress (GO:0080134)	3.12	1.10E-02
mRNA splicing, via spliceosome (GO:0000398)	3.06	1.77E-03
cellular response to lipid (GO:0071396)	2.91	2.76E-02
response to temperature stimulus (GO:0009266)	2.87	1.09E-02
response to oxygen-containing compound (GO:1901700)	2.85	9.22E-06
RNA splicing, via transesterification reactions with bulged adenosine as nucleophile (GO:0000377)	2.84	4.29E-03
RNA splicing, via transesterification reactions (GO:0000375)	2.84	4.23E-03
regulation of hydrolase activity (GO:0051336)	2.72	5.01E-03
small molecule catabolic process (GO:0044282)	2.71	1.82E-02
ribosome biogenesis (GO:0042254)	2.69	1.18E-04
microtubule-based process (GO:0007017)	2.63	2.45E-02
RNA splicing (GO:0008380)	2.58	4.92E-03
cellular response to oxygen-containing compound (GO:1901701)	2.54	4.46E-02
translation (GO:0006412)	2.54	2.75E-06
cell differentiation (GO:0030154)	2.53	3.34E-02
peptide biosynthetic process (GO:0043043)	2.49	4.70E-06
rRNA processing (GO:0006364)	2.48	1.82E-02
rRNA metabolic process (GO:0016072)	2.43	2.01E-02
amide biosynthetic process (GO:0043604)	2.42	3.12E-06
ribonucleoprotein complex biogenesis (GO:0022613)	2.39	3.85E-04
regulation of response to stimulus (GO:0048583)	2.38	1.82E-02

peptide metabolic process (GO:0006518)	2.33	4.85E-06
mRNA processing (GO:0006397)	2.31	9.09E-03
response to abiotic stimulus (GO:0009628)	2.27	6.33E-04
cellular amide metabolic process (GO:0043603)	2.24	4.23E-06
positive regulation of RNA biosynthetic process (GO:1902680)	2.16	2.63E-02
positive regulation of transcription, DNA-templated (GO:0045893)	2.16	2.61E-02
positive regulation of nucleic acid-templated transcription (GO:1903508)	2.16	2.58E-02
negative regulation of nitrogen compound metabolic process (GO:0051172)	2.08	2.85E-02
gene expression (GO:0010467)	2.05	2.99E-09
response to hormone (GO:0009725)	2.04	8.83E-03
response to endogenous stimulus (GO:0009719)	2.02	9.09E-03
mRNA metabolic process (GO:0016071)	2.01	1.78E-02

Supplementary Table S3. GO terms belonging to the "biological process" category of up-regulated genes in the embryo GJ45 (fold enrichment > 2, FDR < 0.05)

GO term	Fold enrichment	FDR
viral process (GO:0016032)	9.98	7.75E-03
nucleosome assembly (GO:0006334)	8.71	2.15E-06
auxin metabolic process (GO:0009850)	7.94	7.11E-03
nucleosome organization (GO:0034728)	7.32	2.04E-06
chromatin assembly (GO:0031497)	6.81	2.25E-05
chromatin assembly or disassembly (GO:0006333)	6.77	9.52E-06
DNA packaging (GO:0006323)	6.04	4.88E-05
biological process involved in symbiotic interaction (GO:0044403)	5.64	2.86E-02
cellular response to topologically incorrect protein (GO:0035967)	5.5	5.01E-04
response to topologically incorrect protein (GO:0035966)	5.32	6.12E-04
cellular response to unfolded protein (GO:0034620)	5.12	2.38E-02
protein refolding (GO:0042026)	4.99	2.51E-02
response to unfolded protein (GO:0006986)	4.87	2.77E-02
response to heat (GO:0009408)	4.66	1.60E-04
protein-DNA complex assembly (GO:0065004)	4.39	4.90E-04
chaperone cofactor-dependent protein refolding (GO:0051085)	4.32	0.0284
'de novo' posttranslational protein folding (GO:0051084)	4.32	2.80E-02
disaccharide metabolic process (GO:0005984)	4.16	7.00E-03
SCF-dependent proteasomal ubiquitin-dependent protein catabolic process (GO:0031146)	4.16	1.37E-03
'de novo' protein folding (GO:0006458)	4.08	3.54E-02
protein-DNA complex subunit organization (GO:0071824)	4.05	4.01E-04
oligosaccharide metabolic process (GO:0009311)	3.61	1.89E-02
ribosome assembly (GO:0042255)	3.57	2.88E-02
response to temperature stimulus (GO:0009266)	3.52	1.11E-04
response to acid chemical (GO:0001101)	3.52	3.11E-02
nuclear transport (GO:0051169)	3.49	9.05E-03
nucleocytoplasmic transport (GO:0006913)	3.49	8.92E-03
proteasomal protein catabolic process (GO:0010498)	3.05	1.11E-04
protein folding (GO:0006457)	3.04	5.01E-04
DNA conformation change (GO:0071103)	3.03	9.00E-03
chromatin organization (GO:0006325)	2.87	2.16E-04
organelle assembly (GO:0070925)	2.74	1.67E-02
proteasome-mediated ubiquitin-dependent protein catabolic process (GO:0043161)	2.64	4.01E-03
response to abiotic stimulus (GO:0009628)	2.58	3.58E-06
modification-dependent protein catabolic process (GO:0019941)	2.45	3.30E-05
modification-dependent macromolecule catabolic process (GO:0043632)	2.4	4.09E-05
proteolysis involved in cellular protein catabolic process (GO:0051603)	2.33	3.41E-05
cellular protein catabolic process (GO:0044257)	2.33	3.34E-05
protein catabolic process (GO:0030163)	2.3	3.95E-05
ubiquitin-dependent protein catabolic process (GO:0006511)	2.25	5.66E-04
RNA splicing (GO:0008380)	2.24	3.57E-02
mRNA processing (GO:0006397)	2.24	7.60E-03
protein-containing complex assembly (GO:0065003)	2.18	1.67E-03
mRNA metabolic process (GO:0016071)	2.18	1.63E-03
cellular macromolecule catabolic process (GO:0044265)	2.16	2.55E-05
cellular protein-containing complex assembly (GO:0034622)	2.16	5.05E-03

response to oxygen-containing compound (GO:1901700)	2.15	7.32E-03
organonitrogen compound catabolic process (GO:1901565)	2.13	4.04E-05
cellular component assembly (GO:0022607)	2.08	4.66E-04
chromosome organization (GO:0051276)	2.08	7.18E-03
cellular response to stress (GO:0033554)	2.06	1.07E-03
protein-containing complex subunit organization (GO:0043933)	2.05	2.55E-03
cellular response to organic substance (GO:0071310)	2.03	2.12E-02
response to organic substance (GO:0010033)	2.01	1.08E-03

Supplementary Table S4. GO terms belonging to the “biological process” category of down-regulated genes in the embryo GJ45 (fold enrichment > 2, FDR < 0.05)

GO term	Fold enrichment	FDR
UDP-glucose transmembrane transport (GO:0015786)	11.54	3.86E-02
photosystem II assembly (GO:0010207)	11.54	3.83E-02
regulation of jasmonic acid mediated signaling pathway (GO:2000022)	8.31	1.48E-03
coenzyme A metabolic process (GO:0015936)	8.11	3.43E-02
response to wounding (GO:0009611)	7.69	8.25E-07
pyrimidine nucleotide-sugar transmembrane transport (GO:0090481)	6.27	1.52E-02
cellular response to abscisic acid stimulus (GO:0071215)	5.27	2.13E-04
cellular response to alcohol (GO:0097306)	5.27	2.09E-04
nucleotide-sugar transmembrane transport (GO:0015780)	5.19	3.35E-02
abscisic acid-activated signaling pathway (GO:0009738)	5.11	5.76E-04
regulation of defense response (GO:0031347)	4.6	7.12E-04
pyrimidine-containing compound biosynthetic process (GO:0072528)	4.51	3.29E-02
purine nucleoside bisphosphate metabolic process (GO:0034032)	4.33	1.27E-02
ribonucleoside bisphosphate metabolic process (GO:0033875)	4.33	1.25E-02
nucleoside bisphosphate metabolic process (GO:0033865)	4.33	1.24E-02
peptidyl-serine phosphorylation (GO:0018105)	3.52	2.64E-02
peptidyl-serine modification (GO:0018209)	3.48	2.87E-02
regulation of response to stress (GO:0080134)	3.39	1.51E-03
protein transmembrane transport (GO:0071806)	3.31	2.53E-02
export from cell (GO:0140352)	3.24	1.93E-02
cellular response to lipid (GO:0071396)	3.2	4.25E-03
plastid organization (GO:0009657)	3.11	3.68E-03
microtubule-based process (GO:0007017)	3.07	8.97E-04
negative regulation of hydrolase activity (GO:0051346)	3.07	2.87E-02
fatty acid metabolic process (GO:0006631)	3.05	1.47E-03
regulation of signal transduction (GO:0009966)	2.99	1.61E-02
chloroplast organization (GO:0009658)	2.98	2.48E-02
regulation of signaling (GO:0023051)	2.97	1.72E-02
protein autophosphorylation (GO:0046777)	2.96	3.51E-02
regulation of cell communication (GO:0010646)	2.93	1.95E-02
fatty acid biosynthetic process (GO:0006633)	2.81	4.94E-02
cytoskeleton organization (GO:0007010)	2.76	3.26E-03
cellular response to oxygen-containing compound (GO:1901701)	2.72	9.80E-03
response to abscisic acid (GO:0009737)	2.72	3.38E-02
response to alcohol (GO:0097305)	2.67	3.87E-02
protein dephosphorylation (GO:0006470)	2.6	1.55E-02
regulation of hydrolase activity (GO:0051336)	2.57	7.32E-03
translation (GO:0006412)	2.56	2.58E-07
ribose phosphate metabolic process (GO:0019693)	2.52	6.97E-03
peptide biosynthetic process (GO:0043043)	2.51	5.46E-07
amide biosynthetic process (GO:0043604)	2.51	9.53E-08
ribonucleotide metabolic process (GO:0009259)	2.5	9.74E-03
cellular amide metabolic process (GO:0043603)	2.38	3.23E-08
peptide metabolic process (GO:0006518)	2.37	5.64E-07
regulation of response to stimulus (GO:0048583)	2.35	1.24E-02
protein localization to organelle (GO:0033365)	2.34	9.79E-03
cellular lipid metabolic process (GO:0044255)	2.34	3.63E-06

nucleoside phosphate metabolic process (GO:0006753)	2.34	6.87E-03
purine ribonucleotide metabolic process (GO:0009150)	2.33	4.47E-02
response to oxygen-containing compound (GO:1901700)	2.3	1.49E-03
nucleobase-containing small molecule metabolic process (GO:0055086)	2.23	4.02E-03
nucleotide metabolic process (GO:0009117)	2.21	2.17E-02
anion transmembrane transport (GO:0098656)	2.2	1.10E-02
monocarboxylic acid metabolic process (GO:0032787)	2.19	7.58E-03
intracellular signal transduction (GO:0035556)	2.19	4.48E-02
lipid biosynthetic process (GO:0008610)	2.15	1.13E-03
carbohydrate derivative biosynthetic process (GO:1901137)	2.14	7.54E-03
organonitrogen compound biosynthetic process (GO:1901566)	2.13	5.53E-10
cellular amino acid metabolic process (GO:0006520)	2.13	9.84E-03
oxoacid metabolic process (GO:0043436)	2.08	2.58E-05
carboxylic acid metabolic process (GO:0019752)	2.08	3.21E-05
cellular response to hormone stimulus (GO:0032870)	2.08	3.45E-02
organic acid metabolic process (GO:0006082)	2.08	1.31E-05
cellular response to endogenous stimulus (GO:0071495)	2.05	3.84E-02
carbohydrate derivative metabolic process (GO:1901135)	2.05	7.70E-04
hormone-mediated signaling pathway (GO:0009755)	2.05	4.79E-02
organophosphate metabolic process (GO:0019637)	2.04	1.03E-03
protein localization (GO:0008104)	2.03	6.75E-05
cellular nitrogen compound biosynthetic process (GO:0044271)	2.01	1.01E-07
small molecule metabolic process (GO:0044281)	2.01	3.66E-08
intracellular protein transport (GO:0006886)	2.01	5.96E-03
protein transport (GO:0015031)	2.01	2.48E-04
cellular macromolecule localization (GO:0070727)	2.01	2.09E-03

Supplementary Table S5. GO terms belonging to the "biological process" category of up-regulated genes in the embryo GJ60 (fold enrichment > 2, FDR < 0.05)

GO term	Fold enrichment	FDR
lipid storage (GO:0019915)	12.43	1.68E-03
sucrose catabolic process (GO:0005987)	8.88	4.92E-03
oligosaccharide catabolic process (GO:0009313)	6.9	1.22E-02
disaccharide catabolic process (GO:0046352)	6.9	1.21E-02
sucrose metabolic process (GO:0005985)	6.83	4.40E-05
protein insertion into mitochondrial inner membrane (GO:0045039)	6.27	3.84E-02
chromatin silencing (GO:0006342)	5.4	3.18E-02
protein complex oligomerization (GO:0051259)	4.99	1.22E-02
disaccharide metabolic process (GO:0005984)	4.93	3.50E-05
oligosaccharide metabolic process (GO:0009311)	4.28	1.48E-04
regulation of gene expression, epigenetic (GO:0040029)	4.18	6.66E-03
maintenance of location (GO:0051235)	4.1	3.28E-02
protein targeting to mitochondrion (GO:0006626)	3.66	9.93E-03
chromatin assembly (GO:0031497)	3.55	2.69E-02
protein localization to mitochondrion (GO:0070585)	3.5	1.26E-02
establishment of protein localization to mitochondrion (GO:0072655)	3.5	1.25E-02
response to salt stress (GO:0009651)	3.47	2.21E-03
nucleosome organization (GO:0034728)	3.37	3.73E-02
response to osmotic stress (GO:0006970)	3.33	9.63E-04
chromatin assembly or disassembly (GO:0006333)	3.31	4.12E-02
positive regulation of gene expression (GO:0010628)	3.28	2.87E-02
response to water deprivation (GO:0009414)	3.25	2.08E-02
response to cold (GO:0009409)	3.25	1.02E-02
response to temperature stimulus (GO:0009266)	3.24	4.25E-05
negative regulation of transcription, DNA-templated (GO:0045892)	3.19	8.26E-04
negative regulation of RNA biosynthetic process (GO:1902679)	3.17	8.45E-04
negative regulation of nucleic acid-templated transcription (GO:1903507)	3.17	8.22E-04
response to water (GO:0009415)	3.16	2.47E-02
negative regulation of nucleobase-containing compound metabolic process (GO:0045934)	3.13	2.87E-04
response to heat (GO:0009408)	3.12	9.88E-03
protein transmembrane transport (GO:0071806)	3.02	1.20E-02
negative regulation of RNA metabolic process (GO:0051253)	3	1.54E-03
response to acid chemical (GO:0001101)	2.96	3.87E-02
mitochondrial transport (GO:0006839)	2.93	1.47E-02
response to inorganic substance (GO:0010035)	2.81	6.36E-04
protein folding (GO:0006457)	2.77	3.23E-04
response to abscisic acid (GO:0009737)	2.73	4.87E-03
response to alcohol (GO:0097305)	2.68	6.05E-03
mitochondrion organization (GO:0007005)	2.54	9.97E-03
mRNA splicing, via spliceosome (GO:0000398)	2.36	1.37E-02
response to lipid (GO:0033993)	2.26	8.16E-03
chromatin organization (GO:0006325)	2.24	4.67E-03
RNA splicing, via transesterification reactions with bulged adenosine as nucleophile (GO:0000377)	2.2	2.31E-02
RNA splicing, via transesterification reactions (GO:0000375)	2.2	2.29E-02
response to oxygen-containing compound (GO:1901700)	2.18	6.68E-04

establishment of protein localization to organelle (GO:0072594)	2.12	1.62E-02
protein localization to organelle (GO:0033365)	2.12	9.90E-03
translation (GO:0006412)	2.03	1.51E-04
peptide biosynthetic process (GO:0043043)	2.02	1.47E-04
negative regulation of biosynthetic process (GO:0009890)	2.01	4.87E-02

Supplementary Table S6. GO terms belonging to the “biological process” category of down-regulated genes in the embryo GJ60 (fold enrichment > 2, FDR < 0.05)

GO term	Fold enrichment	FDR
cell migration (GO:0016477)	10.88	3.63E-02
DNA ligation (GO:0006266)	10.88	3.62E-02
phospholipid translocation (GO:0045332)	9.07	1.17E-04
transcription-dependent tethering of RNA polymerase II gene DNA at nuclear periphery (GO:0000972)	9.07	7.18E-03
inositol lipid-mediated signaling (GO:0048017)	8.5	2.35E-02
phosphatidylinositol-mediated signaling (GO:0048015)	8.5	2.34E-02
lipid translocation (GO:0034204)	7.56	3.58E-04
glycogen biosynthetic process (GO:0005978)	6.8	4.11E-02
regulation of membrane lipid distribution (GO:0097035)	6.8	6.94E-04
L-phenylalanine catabolic process (GO:0006559)	6.35	1.05E-02
erythrose 4-phosphate/phosphoenolpyruvate family amino acid catabolic process (GO:1902222)	6.35	1.05E-02
methylguanosine-cap decapping (GO:0110156)	6.28	2.45E-02
glycogen metabolic process (GO:0005977)	6.12	2.64E-03
energy reserve metabolic process (GO:0006112)	6.12	2.63E-03
cortical microtubule organization (GO:0043622)	6.04	5.96E-03
vesicle cargo loading (GO:0035459)	5.83	3.14E-02
aromatic amino acid family catabolic process (GO:0009074)	5.83	3.37E-03
RNA decapping (GO:0110154)	5.83	3.13E-02
phospholipid transport (GO:0015914)	5.77	8.36E-05
regulation of cell growth (GO:0001558)	5.44	9.83E-03
organophosphate ester transport (GO:0015748)	5.25	6.38E-07
establishment or maintenance of cell polarity (GO:0007163)	5.1	4.71E-02
cytoplasmic microtubule organization (GO:0031122)	4.99	2.06E-03
branched-chain amino acid biosynthetic process (GO:0009082)	4.9	8.39E-03
spliceosomal complex assembly (GO:0000245)	4.76	3.28E-02
cortical cytoskeleton organization (GO:0030865)	4.69	5.66E-03
phosphatidylinositol phosphorylation (GO:0046854)	4.69	5.63E-03
protein N-linked glycosylation (GO:0006487)	4.65	9.98E-04
receptor-mediated endocytosis (GO:0006898)	4.53	1.18E-03
gene silencing by miRNA (GO:0035195)	4.53	3.90E-02
branched-chain amino acid metabolic process (GO:0009081)	4.37	1.46E-02
response to UV (GO:0009411)	4.35	2.65E-02
vesicle budding from membrane (GO:0006900)	4.11	2.39E-03
L-phenylalanine metabolic process (GO:0006558)	4.08	2.07E-02
erythrose 4-phosphate/phosphoenolpyruvate family amino acid metabolic process (GO:1902221)	4.08	2.06E-02
maturation of LSU-rRNA (GO:0000470)	4.05	1.57E-03
pteridine-containing compound metabolic process (GO:0042558)	4.03	3.65E-02
alpha-amino acid catabolic process (GO:1901606)	4.03	6.00E-04
endocytosis (GO:0006897)	4.02	3.74E-05
nucleotide-sugar metabolic process (GO:0009225)	3.89	5.85E-03
lipid phosphorylation (GO:0046834)	3.89	1.61E-02
clathrin-dependent endocytosis (GO:0072583)	3.89	4.17E-02
transcription by RNA polymerase II (GO:0006366)	3.78	1.38E-04
one-carbon metabolic process (GO:0006730)	3.71	3.28E-02
cellular amino acid catabolic process (GO:0009063)	3.71	4.80E-04

starch metabolic process (GO:0005982)	3.68	5.25E-03
RNA 3'-end processing (GO:0031123)	3.68	4.16E-05
lipid oxidation (GO:0034440)	3.63	9.34E-03
transcription initiation from RNA polymerase II promoter (GO:0006367)	3.58	2.50E-02
regulation of mRNA metabolic process (GO:1903311)	3.56	1.64E-02
posttranscriptional gene silencing (GO:0016441)	3.54	7.04E-03
ribosomal large subunit biogenesis (GO:0042273)	3.54	1.74E-05
nucleotide-sugar transmembrane transport (GO:0015780)	3.5	4.21E-02
post-transcriptional gene silencing by RNA (GO:0035194)	3.47	1.20E-02
supramolecular fiber organization (GO:0097435)	3.37	1.48E-05
regulation of phosphatase activity (GO:0010921)	3.34	4.55E-03
import into nucleus (GO:0051170)	3.32	2.45E-02
movement of cell or subcellular component (GO:0006928)	3.31	1.48E-03
regulation of protein dephosphorylation (GO:0035304)	3.28	7.89E-03
regulation of phosphoprotein phosphatase activity (GO:0043666)	3.28	7.86E-03
chromatin remodeling (GO:0006338)	3.26	1.83E-02
endosomal transport (GO:0016197)	3.24	5.77E-03
rRNA modification (GO:0000154)	3.24	4.11E-02
dsRNA processing (GO:0031050)	3.24	4.10E-02
production of small RNA involved in gene silencing by RNA (GO:0070918)	3.24	4.08E-02
maturation of 5.8S rRNA (GO:0000460)	3.24	4.07E-02
carboxylic acid catabolic process (GO:0046395)	3.21	1.16E-04
regulation of dephosphorylation (GO:0035303)	3.19	6.58E-03
gene silencing by RNA (GO:0031047)	3.16	1.07E-03
lipid modification (GO:0030258)	3.12	4.95E-05
nuclear export (GO:0051168)	3.12	1.11E-02
vacuole organization (GO:0007033)	3.05	3.90E-02
RNA export from nucleus (GO:0006405)	3.05	1.95E-02
membrane organization (GO:0061024)	3.01	5.45E-08
positive regulation of catalytic activity (GO:0043085)	3	9.56E-05
histone methylation (GO:0016571)	2.99	4.26E-02
nuclear transport (GO:0051169)	2.99	9.40E-04
nucleocytoplasmic transport (GO:0006913)	2.99	9.33E-04
carbohydrate derivative transport (GO:1901264)	2.96	5.75E-03
vesicle organization (GO:0016050)	2.94	1.55E-03
positive regulation of GTPase activity (GO:0043547)	2.93	4.77E-02
positive regulation of molecular function (GO:0044093)	2.89	1.68E-04
phosphatidylinositol metabolic process (GO:0046488)	2.89	1.91E-03
organic acid catabolic process (GO:0016054)	2.88	5.15E-04
peptidyl-lysine methylation (GO:0018022)	2.86	3.97E-02
aromatic amino acid family metabolic process (GO:0009072)	2.83	1.18E-02
sulfur compound biosynthetic process (GO:0044272)	2.8	2.62E-03
actin filament organization (GO:0007015)	2.79	1.82E-02
actin cytoskeleton organization (GO:0030036)	2.79	1.31E-02
actin filament-based process (GO:0030029)	2.79	1.30E-02
vacuolar transport (GO:0007034)	2.75	4.38E-03
gene silencing (GO:0016458)	2.74	1.73E-03
nucleobase-containing compound transport (GO:0015931)	2.72	2.23E-04
microtubule cytoskeleton organization (GO:0000226)	2.7	1.57E-03
cytoskeleton organization (GO:0007010)	2.69	8.47E-06
positive regulation of hydrolase activity (GO:0051345)	2.69	1.81E-02

establishment of RNA localization (GO:0051236)	2.65	1.97E-02
RNA transport (GO:0050658)	2.65	1.97E-02
nucleic acid transport (GO:0050657)	2.65	1.96E-02
rRNA metabolic process (GO:0016072)	2.63	2.38E-06
protein localization to membrane (GO:0072657)	2.6	1.20E-03
rRNA processing (GO:0006364)	2.57	6.05E-06
microtubule-based process (GO:0007017)	2.56	1.35E-04
histone modification (GO:0016570)	2.54	2.00E-03
covalent chromatin modification (GO:0016569)	2.54	1.99E-03
RNA localization (GO:0006403)	2.54	2.85E-02
ncRNA processing (GO:0034470)	2.48	2.23E-08
glycerophospholipid metabolic process (GO:0006650)	2.46	2.88E-03
cellular response to DNA damage stimulus (GO:0006974)	2.45	3.88E-07
ncRNA metabolic process (GO:0034660)	2.45	8.39E-10
phospholipid biosynthetic process (GO:0008654)	2.45	8.55E-03
chromatin organization (GO:0006325)	2.45	1.48E-05
negative regulation of gene expression (GO:0010629)	2.45	6.37E-06
ribosome biogenesis (GO:0042254)	2.43	2.69E-07
small molecule catabolic process (GO:0044282)	2.42	9.46E-04
RNA processing (GO:0006396)	2.41	7.05E-16
protein glycosylation (GO:0006486)	2.41	5.03E-03
macromolecule glycosylation (GO:0043413)	2.41	5.01E-03
phospholipid metabolic process (GO:0006644)	2.4	2.88E-04
glycoprotein biosynthetic process (GO:0009101)	2.39	5.19E-03
DNA repair (GO:0006281)	2.38	2.74E-06
fatty acid metabolic process (GO:0006631)	2.36	1.56E-03
ribonucleoprotein complex biogenesis (GO:0022613)	2.35	2.24E-08
alpha-amino acid metabolic process (GO:1901605)	2.35	1.16E-04
protein import (GO:0017038)	2.35	2.61E-02
glycoprotein metabolic process (GO:0009100)	2.34	4.69E-03
RNA catabolic process (GO:0006401)	2.32	2.40E-02
organelle assembly (GO:0070925)	2.32	3.86E-03
organelle organization (GO:0006996)	2.3	1.78E-23
chromosome organization (GO:0051276)	2.29	1.85E-07
glycerolipid metabolic process (GO:0046486)	2.28	7.48E-03
lipid transport (GO:0006869)	2.27	2.68E-02
regulation of hydrolase activity (GO:0051336)	2.27	7.52E-04
hexose metabolic process (GO:0019318)	2.27	3.45E-02
DNA replication (GO:0006260)	2.25	2.18E-02
DNA metabolic process (GO:0006259)	2.25	3.51E-07
RNA phosphodiester bond hydrolysis, endonucleolytic (GO:0090502)	2.23	2.97E-02
monocarboxylic acid biosynthetic process (GO:0072330)	2.23	9.87E-03
cellular localization (GO:0051641)	2.21	1.44E-12
mRNA metabolic process (GO:0016071)	2.2	4.33E-07
intracellular transport (GO:0046907)	2.2	3.61E-10
cellular amino acid metabolic process (GO:0006520)	2.19	8.80E-06
mRNA processing (GO:0006397)	2.19	3.74E-05
macromolecule methylation (GO:0043414)	2.19	1.08E-02
establishment of localization in cell (GO:0051649)	2.18	2.47E-10
organic cyclic compound catabolic process (GO:1901361)	2.18	1.18E-03
RNA phosphodiester bond hydrolysis (GO:0090501)	2.18	5.00E-03
aromatic compound catabolic process (GO:0019439)	2.17	1.57E-03

alpha-amino acid biosynthetic process (GO:1901607)	2.15	1.96E-02
protein phosphopantetheinylation (GO:0018215)	2.13	4.71E-03
cellular response to stress (GO:0033554)	2.13	5.81E-08
cellular protein localization (GO:0034613)	2.12	6.50E-08
heterocycle catabolic process (GO:0046700)	2.11	9.81E-03
posttranscriptional regulation of gene expression (GO:0010608)	2.11	2.17E-03
RNA metabolic process (GO:0016070)	2.11	4.54E-19
cellular macromolecule localization (GO:0070727)	2.1	8.19E-08
nucleic acid phosphodiester bond hydrolysis (GO:0090305)	2.1	3.50E-04
cellular nitrogen compound catabolic process (GO:0044270)	2.09	1.05E-02
nucleic acid metabolic process (GO:0090304)	2.09	1.27E-24
intracellular protein transport (GO:0006886)	2.08	1.17E-06
monocarboxylic acid metabolic process (GO:0032787)	2.08	1.05E-04
RNA splicing (GO:0008380)	2.07	2.89E-03
organic substance transport (GO:0071702)	2.05	1.03E-14
carboxylic acid metabolic process (GO:0019752)	2.04	1.63E-09
cellular component organization or biogenesis (GO:0071840)	2.04	5.15E-29
DNA recombination (GO:0006310)	2.03	4.73E-02
cellular component organization (GO:0016043)	2.03	1.58E-24
transcription, DNA-templated (GO:0006351)	2.02	3.45E-03
carboxylic acid biosynthetic process (GO:0046394)	2.02	1.66E-04
oxoacid metabolic process (GO:0043436)	2.01	3.53E-09
cellular lipid metabolic process (GO:0044255)	2.01	2.92E-07
negative regulation of macromolecule metabolic process (GO:0010605)	2.01	4.25E-06
vesicle-mediated transport (GO:0016192)	2.01	2.26E-05

Supplementary Table S7. GO terms belonging to the "biological process" category of up-regulated genes in the endosperm GJ45 (fold enrichment > 2, FDR < 0.05)

GO term	Fold enrichment	FDR
regulation of protein serine/threonine phosphatase activity (GO:0080163)	52.69	1.36E-02
negative regulation of protein dephosphorylation (GO:0035308)	37.39	2.83E-02
negative regulation of phosphoprotein phosphatase activity (GO:0032515)	37.39	2.67E-02
negative regulation of phosphatase activity (GO:0010923)	36.22	2.75E-02
negative regulation of dephosphorylation (GO:0035305)	36.22	2.61E-02
negative regulation of hydrolase activity (GO:0051346)	21.07	9.74E-04
negative regulation of catalytic activity (GO:0043086)	13.86	8.12E-04
negative regulation of molecular function (GO:0044092)	13.8	4.20E-04
negative regulation of cellular protein metabolic process (GO:0032269)	12.58	2.30E-03
negative regulation of protein metabolic process (GO:0051248)	12.58	1.84E-03
regulation of hydrolase activity (GO:0051336)	10.44	1.19E-02
negative regulation of cellular metabolic process (GO:0031324)	7.75	6.08E-03
negative regulation of nitrogen compound metabolic process (GO:0051172)	7.25	1.97E-02
regulation of cellular protein metabolic process (GO:0032268)	7.01	5.57E-03
regulation of protein metabolic process (GO:0051246)	6.66	6.23E-03
regulation of catalytic activity (GO:0050790)	6.5	6.04E-03
regulation of molecular function (GO:0065009)	6.42	6.07E-03
negative regulation of cellular process (GO:0048523)	6.11	2.04E-02
defense response (GO:0006952)	5.92	6.24E-03
negative regulation of metabolic process (GO:0009892)	5.51	2.88E-02
response to stress (GO:0006950)	3.22	2.78E-02

Supplementary Table S8. GO terms belonging to the "biological process" category of down-regulated genes in the endosperm GJ45 (fold enrichment > 2, FDR < 0.05)

GO term	Fold enrichment	FDR
glycogen biosynthetic process (GO:0005978)	> 100	1.56E-02
glycogen metabolic process (GO:0005977)	65.49	4.76E-02
energy reserve metabolic process (GO:0006112)	65.49	3.17E-02

Supplementary Table S9. GO terms belonging to the "biological process" category of up-regulated genes in the endosperm GJ60 (fold enrichment > 2, FDR < 0.05)

GO term	Fold enrichment	FDR
response to ethanol (GO:0045471)	> 100	1.52E-02
toxin metabolic process (GO:0009404)	> 100	3.01E-02
toxin biosynthetic process (GO:0009403)	> 100	2.87E-02
phytoalexin biosynthetic process (GO:0052315)	> 100	2.74E-02
phytoalexin metabolic process (GO:0052314)	> 100	2.62E-02
response to arsenic-containing substance (GO:0046685)	> 100	2.51E-02
diterpene phytoalexin biosynthetic process (GO:0051502)	> 100	2.41E-02
diterpene phytoalexin metabolic process (GO:0051501)	> 100	2.32E-02
response to copper ion (GO:0046688)	> 100	2.97E-02
diterpenoid biosynthetic process (GO:0016102)	50.53	4.51E-04
diterpenoid metabolic process (GO:0016101)	40.43	4.20E-04
negative regulation of hydrolase activity (GO:0051346)	22.05	4.48E-04
terpenoid biosynthetic process (GO:0016114)	21.66	4.13E-04
negative regulation of endopeptidase activity (GO:0010951)	21.56	1.56E-02
negative regulation of peptidase activity (GO:0010466)	21.56	1.46E-02
negative regulation of proteolysis (GO:0045861)	21.56	1.37E-02
regulation of endopeptidase activity (GO:0052548)	21.28	1.35E-02
regulation of peptidase activity (GO:0052547)	21	1.34E-02
terpenoid metabolic process (GO:0006721)	18.95	7.47E-04
isoprenoid biosynthetic process (GO:0008299)	18.74	3.44E-04
isoprenoid metabolic process (GO:0006720)	15.99	4.84E-04
negative regulation of cellular protein metabolic process (GO:0032269)	11.28	1.16E-02
negative regulation of protein metabolic process (GO:0051248)	11.28	1.03E-02
regulation of hydrolase activity (GO:0051336)	10.93	1.10E-02
negative regulation of catalytic activity (GO:0043086)	10.88	1.03E-02
negative regulation of molecular function (GO:0044092)	10.83	9.65E-03
lipid biosynthetic process (GO:0008610)	6.55	1.35E-02
response to stress (GO:0006950)	3.13	4.78E-02
